# A novel aqueous extract from rice fermented with *Aspergillus oryzae* and *Saccharomyces cerevisiae* possesses an anti-influenza A virus activity

**DOI:** 10.1371/journal.pone.0244885

**Published:** 2021-01-15

**Authors:** Masaki Shoji, Minami Sugimoto, Kosuke Matsuno, Yoko Fujita, Tomohiro Mii, Satomi Ayaki, Misa Takeuchi, Saki Yamaji, Narue Tanaka, Etsuhisa Takahashi, Takeshi Noda, Hiroshi Kido, Takaaki Tokuyama, Takahito Tokuyama, Takashi Tokuyama, Takashi Kuzuhara

**Affiliations:** 1 Faculty of Pharmaceutical Sciences, Laboratory of Biochemistry, Tokushima Bunri University, Yamashiro-cho, Tokushima, Japan; 2 Yushin Brewer Co. Ltd., Ono, Ayagawa-cho, Ayauta-gun, Kagawa, Japan; 3 Laboratory of Ultrastructural Virology, Graduate School of Biostudies, Kyoto University, Shogoin Kawahara-cho, Sakyo-ku, Kyoto, Japan; 4 Laboratory of Ultrastructural Virology, Institute for Frontier Life and Medical Sciences, Kyoto University, Shogoin Kawahara-cho, Sakyo-ku, Kyoto, Japan; 5 Division of Pathology and Metabolome Research for Infectious Disease and Host Defense, Institute for Enzyme Research, University of Tokushima, Kuramoto-cho, Tokushima, Japan; University of Washington, UNITED STATES

## Abstract

Human influenza virus infections occur annually worldwide and are associated with high morbidity and mortality. Hence, development of novel anti-influenza drugs is urgently required. Rice Power^®^ extract developed by the Yushin Brewer Co. Ltd. is a novel aqueous extract of rice obtained via saccharization and fermentation with various microorganisms, such as *Aspergillus oryzae*, yeast [such as *Saccharomyces cerevisiae*], and lactic acid bacteria, possessing various biological and pharmacological properties. In our previous experimental screening with thirty types of Rice Power^®^ extracts, we observed that the 30^th^ Rice Power^®^ (Y30) extract promoted the survival of influenza A virus-infected Madin-Darby canine kidney (MDCK) cells. Therefore, to identify compounds for the development of novel anti-influenza drugs, we aimed to investigate whether the Y30 extract exhibits anti-influenza A virus activity. In the present study, we demonstrated that the Y30 extract strongly promoted the survival of influenza A H1N1 Puerto Rico 8/34 (A/PR/8/34), California 7/09, or H3N2 Aichi 2/68 (A/Aichi/2/68) viruses-infected MDCK cells and inhibited A/PR/8/34 or A/Aichi/2/68 viruses infection and growth in the co-treatment and pre-infection experiments. The pre-treatment of Y30 extract on MDCK cells did not induce anti-influenza activity in the cell. The Y30 extract did not significantly affect influenza A virus hemagglutination, and neuraminidase and RNA-dependent RNA polymerase activities. Interestingly, the electron microscopy experiment revealed that the Y30 extract disrupts the integrity of influenza A virus particles by permeabilizing the viral membrane envelope, suggesting that Y30 extract has a direct virucidal effect against influenza A virus. Furthermore, we observed that compared to the ethyl acetate (EtOAc) extract, the water extract of Y30 extract considerably promoted the survival of cells infected with A/PR/8/34 virus. These results indicated that more anti-influenza components were present in the water extract of Y30 extract than in the EtOAc extract. Our results highlight the potential of a rice extract fermented with *A*. *oryzae* and *S*. *cerevisiae* as an anti-influenza medicine and a drug source for the development of anti-influenza compounds.

## Introduction

The World Health Organization Fact sheet shows that approximately 3–5 million cases of severe influenza infection occur annually, out of which approximately 290,000 to 650,000 cases result in mortality [1]. Two classes of anti-influenza virus drugs are currently available: neuraminidase (NA) inhibitors, such as oseltamivir, zanamivir, and peramivir, and M2 proton channel inhibitors, such as amantadine and rimantadine of the adamantane family of antiviral drugs. Considering that adamantane-resistant influenza strains have been frequently reported [1–4], the use of NA inhibitors is currently recommended for influenza treatment. However, oseltamivir-resistant influenza strains were detected in some influenza A H1N1 viruses in 2009 and in the seasonal H1N1 viruses between 2007 and 2009, although the prevalence of drug-resistant influenza A H3N2 and H5N1 viruses is low [2,5–7]. In addition, baloxavir marboxil, which inhibits the cap-dependent endonuclease activity of influenza polymerase acidic (PA) protein, is a novel antiviral drug against influenza A and B strains and was approved in 2018 [8]. However, baloxavir-resistant influenza A H1N1 and H3N2 viruses with I38T-substituted PA have been detected [9], and the H3N2 virus with PA-I38T substitution can be transmitted among humans [10]. Therefore, development of novel anti-influenza drugs for preventing and controlling potential influenza epidemics and pandemics is urgently required.

Fermented rice extracts have been reported to exhibit various biological and pharmacological properties, including prevention of NaCl or methotrexate cytotoxicity in the gastric mucosa [11,12], inhibition of *Helicobacter pylori* (*H*. *pylori*) infection [13], anti-oxidant activity [14–17], anti-inflammatory activity in the intestinal mucosa [18] or smooth muscle [19], anti-tumor [20–22] and anti-leukemia properties [23], anti-neurotoxic activity in Alzheimer’s or Parkinson’s diseases [24,25], blood and hepatic cholesterol-lowering and anti-steatosis activities [26], and enhancement of lipid metabolism [27]. *Aspergillus oryzae* (*A*. *oryzae*)-fermented rice extracts possess anti-inflammatory [13] and anti-oxidant activities [17], which have been reported to be related to anti-influenza virus activity [28–34]. However, whether *A*. *oryzae*-fermented rice extracts possess anti-influenza A virus activity remains unclear.

Rice Power^®^ extract developed by Yushin Brewer Co. Ltd. is a novel aqueous extract of rice obtained via saccharization and fermentation with various microorganisms, such as *A*. *oryzae*, yeast [such as *Saccharomyces cerevisiae* (*S*. *cerevisiae*)], and lactic acid bacteria. It can be produced from various aqueous rice extracts by altering the type and number of bacteria and the fermentation conditions. Currently, 32 types of Rice Power^®^ extract are available. These extracts perform biological and pharmacological activities; for example, they act as insulators and can be used in skin care products, can prevent NaCl-induced histopathological damage and cell proliferation [11], inhibit inflammation and epithelial cell proliferation during *H*. *pylori* infection [13] in the gastric mucosa, enhance barrier and water retention functions, and reduce cutaneous sebum secretion. Hence, we have previously screened 30 types of Rice Power^®^ extracts to analyze their anti-influenza A virus activities and observed that the 30^th^ Rice Power^®^, namely the Y30 extract, promoted the survival of influenza A virus-infected Madin-Darby canine kidney (MDCK) cells. Therefore, we aimed to examine in detail the anti-influenza A virus activity of the Y30 extract in this study.

## Materials and methods

### Production of the aqueous Y30 rice extract

The Y30 extract, the 30^th^ Rice Power^®^ extract, was obtained from Yushin Brewer. Co Ltd. (Kagawa, Japan). The extract was prepared by concentrating the culture filtrate produced after saccharization of rice using *A*. *oryzae*, followed by fermentation using *S*. *cerevisiae* and heating at 85°C. The pH of the Y30 extract was 6.50 and specific gravity was 1.05, and it did not contain ethanol. The rate of evaporation of residues from the extract was 6.8%.

### Preparing of water or ethyl acetate (EtOAc) extracts of Y30 extract

The Y30 extract was added to EtOAc (Wako, Osaka, Japan). Once the extract separated into water and EtOAc layers after standing, the EtOAc layer was collected, and the steps were repeated. The isolated water or EtOAc layers were concentrated using a rotary evaporator under reduced pressure. The dried extracts were dissolved in water, and water or EtOAc extracts were obtained. Thus, EtOAc was not present in the extract because the EtOAc layer was dried completely.

### Cell culture

MDCK cells were cultured in high-glucose Dulbecco’s modified Eagle’s medium (DMEM; Wako) supplemented with 10% fetal bovine serum (FBS; Thermo Fisher Scientific, MA, USA), 100 units/mL penicillin and 100 μg/mL streptomycin (P/S; Thermo Fisher Scientific), and 4 mM l-glutamine at 37°C in the presence of 5% CO_2_.

### Viral strain

The Puerto Rico 8/34 (A/PR/8/34; H1N1), California 7/09 [A/CA/7/09; H1N1, 2009 pandemic strain (H1N1pdm09)], and Aichi 2/68 (A/Aichi/2/68; H3N2) strains of the influenza A viruses [34] were used for the experiments. Viral titers were determined by immunostaining the influenza A viral nucleoprotein (NP) as described previously [34,35].

### Thiazolyl blue tetrazolium bromide (MTT) assay

The MDCK cells were seeded on a 96-well plate at the density of 1 × 10^4^ cells/well. Y30 extract (final 0.4–25%) was prepared in water (final 0.4–25%) and mixed with infection medium [DMEM supplemented with 1% bovine serum albumin (BSA; Wako), P/S, and 4 mM l-glutamine]. The resulting mixture was added to the cells and subsequently incubated for 24 or 72 h at 37°C in the presence of 5% CO_2_. After incubation, the cell viability was determined using the MTT cell proliferation assay as described previously [34,36].

### Analysis of the viability of influenza A virus-infected MDCK cells using naphthol blue-black (NB) staining

The MDCK cells were seeded on a 96-well plate (1 × 10^4^ cells/well). The Y30 extract (final 0.8–12.5%) was mixed with A/PR/8/34, A/CA/7/09, or A/Aichi/2/68 viruses in 10% FBS-supplemented growth medium at a multiplicity of infection (MOI) of ten. In addition, the Y30 extract, the Y30 extract of different lots, water or EtOAc extracts from Y30 extract (final 0.8–12.5%) were mixed with the A/PR/8/34 virus in 10% FBS-supplemented growth medium at MOI of ten. The samples were then incubated for 30 min at 37°C in the presence of 5% CO_2_. Water (final 0.8–12.5%) or (+)-(*S*)-bakuchiol (bakuchiol; final 1.6–25 μM) [34] were used as negative or positive controls, respectively. The resulting mixture was added to the cells, which were then incubated for 72 h at 37°C in the presence of 5% CO_2_. After incubation, the cells were stained with NB as described previously [34,36,37]. Viable cells in each well were stained blue, whereas the dead cells remained unstained.

### Immunofluorescence (IF) staining of influenza A virus-infected MDCK cells

The MDCK cells were seeded on a 96-well plate at the density of 1 × 10^4^ cells/well. The Y30 extract (final 3.1–12.5%) was mixed with A/PR/8/34 or A/Aichi/2/68 viruses at a MOI of 0.1 in the infection medium and incubated for 30 min at 37°C in the presence of 5% CO_2_. Each mixture was added to the cells at 37°C in the presence of 5% CO_2_. The cells were treated with the Y30 extract (final 3.1–12.5%) for 24 h at 37°C in the presence of 5% CO_2_. Subsequently, the Y30-treated cells were washed prior to the addition of the A/PR/8/34 virus at a MOI of 0.1 in the infection medium. Water (final 3.1–12.5%) or bakuchiol (final 3.1–12.5 μM) were used as the negative or positive controls, respectively. After influenza A virus infection for 24 h, the cells were fixed with 4% paraformaldehyde in phosphate-buffered saline (PBS) for 30 min at 4°C and subsequently permeabilized with 0.3% Triton X-100 for 20 min at 25°C. A mouse primary antibody was used to detect the NP of A/PR/8/34 or A/Aichi/2/68 viruses (FluA-NP 4F1; SouthernBiotech, AL, USA). An Alexa Fluor 488-conjugated goat anti-mouse IgG (H + L) antibody (Thermo Fisher Scientific) was used as the secondary antibody. Cell nuclei were then stained using diamidino-2-phenylindole (DAPI; Thermo Fisher Scientific). Wells were photographed using a fluorescence microscope (BIOREVO BZ-X700, Keyence, Osaka, Japan). The percentage of influenza A NP-positive cells per DAPI-positive cells were calculated based on measurements recorded using the BZ-X Analyzer software (Keyence).

### Influenza A virus growth assay

The MDCK cells were seeded on a 24-well plate at the density of 1 × 10^5^ cells/well. The cells were infected with A/PR/8/34 or A/Aichi/2/68 viruses at a MOI of 0.001 in infection medium for 1 h at 37°C in the presence of 5% CO_2_. The infected cells were washed prior to the addition of the Y30 extract (final 6.25%) in infection medium supplemented with 3 μg/mL l-tosylamido-2-phenyl ethyl chloromethyl ketone (TPCK)-treated trypsin (Sigma-Aldrich, MO, USA). The cells were treated with the Y30 extract (final 6.25%) for 24 h at 37°C in the presence of 5% CO_2_. Cells were washed prior to the addition of the A/PR/8/34 virus at a MOI of 0.001 in the infection medium for 1 h at 37°C in the presence of 5% CO_2_. Subsequently, the infection medium supplemented with 3 μg/mL TPCK-treated trypsin (Sigma-Aldrich) was added in the wells of Y30-treated and virus-infected cells. Water (final 6.25%) or ribavirin (final 25 μM) [38] were used as the negative or positive controls, respectively. The cells were then incubated for 24, 48, or 72 h at 37°C in the presence of 5% CO_2_. Culture media were collected from each well at the indicated time points. Viral titers [plaque forming units per mL (PFU/mL)] were determined by immunostaining the influenza A viral NP and calculated as described previously [34,36].

Although the collected culture media in the pre-infection experiment contains samples, each medium was diluted more than one hundred times when viral titers were measured. Thus, the determination of viral titer was not affected by the samples included in the media.

### Hemagglutination inhibition (HAI) assay

To determine the HA activities of A/PR/8/34 or A/CA/7/09 viruses, the viruses (final 0.08–2.5%) were serially diluted with PBS in a round-bottom 96-well plate and incubated for 30 min at 37°C in the presence of 5% CO_2_. Rabbit red blood cells (rRBCs, Cosmo Bio, Tokyo, Japan) were added to each well to a concentration of 0.5% in PBS. The plate was photographed after incubation for 1 h at 25°C. To assess the HAI activities, the Y30 extracts (final 0.8–25%) were diluted with PBS in a round-bottom 96-well plate. Water (final 0.8–25%) was used as the negative control. Samples were mixed with A/PR/8/34 or A/CA/7/09 viruses and incubated for 30 min at 37°C in the presence of 5% CO_2_. rRBCs were added to each mixture at the concentration of 0.5% in PBS. Plates were photographed after incubation for 1 h at 25°C.

### Neuraminidase inhibition (NAI) assay with influenza A virus particles

NA assays were performed as described previously [34]. Briefly, Y30 extract (final 3.1–25%) was diluted with assay buffer (50 mM Tris, 5 mM CaCl_2_, and 200 mM NaCl at pH 7.5) in a 96-well black plate (Thermo Fisher Scientific). Water (final 3.1–25%) or oseltamivir carboxylate [39] (final 3.1–25 μM) were used as the negative or positive control, respectively. Each sample was treated with or without A/PR/8/34 or A/CA/7/09 viruses (1 × 10^4^ PFU) in assay buffer and incubated for 30 min at 37°C in the presence of 5% CO_2_. Each sample was then mixed with 12.5 μM 2ʹ-(4-methylumbelliferyl)-α-D-*N*-acetylneuraminic acid (Sigma-Aldrich) in the 96-well plate. After 3 or 24 h at 37°C in the presence of 5% CO_2_, the reaction was monitored using a fluorescence spectrometer in kinetic mode using excitation wavelength of 365 nm and emission wavelength of 445 nm.

### Influenza A virus minigenome assay

A minigenome assay based on the firefly and *Renilla* luciferase systems was performed as described previously [34,40]. The plasmids, pCAGGS (pCA)-PA, pCA-PB1, pCA-PB2, and pCA-NP harbored the influenza virus PA, PB1, PB2, and NP genes, respectively, driven by the cytomegalovirus enhancer fused to the chicken beta-actin promoter. pHH21-PolI/NP(0)Fluc(0) expressed the minus RNA strand of the firefly luciferase driven by the human RNA polymerase I promoter; this can be converted to the plus strand (mRNA) by influenza virus RNA-dependent RNA polymerase (RdRp). The pRL-TK-Rluc vector (Promega, CA, USA) expressing the *Renilla* luciferase gene driven by the herpes simplex viral thymidine kinase promoter was used as an internal control. MDCK cells (5 × 10^4^) were transfected with 0.12 μg each of pCA-PA, -PB1, -PB2, and -NP, pHH21-PolI/NP(0)Fluc(0), and pRL-TK-Rluc. At 6 h post-transfection, the cells were treated with the Y30 extract (final 6.25%) at 37°C in the presence of 5% CO_2_. Water (final 6.25%) or ribavirin (final 25 μM) were used as the negative or positive control, respectively. After 24 h of incubation, firefly and *Renilla* luciferase activities in the transfected MDCK cells were measured using the Dual-Glo luciferase assay system (Promega) according to the manufacturer’s instructions. Luciferase activity was expressed as the relative light unit (RLU), where the activity of the water-treated cells was considered 100%.

### Analysis of the disrupted influenza A virus particles by electron microscopy

The supernatant of MDCK cells infected with A/PR/8/34 virus was collected at three days post-infection. After removal of cell debris by low speed centrifugation, the supernatant was mixed with Y30 extract (final 12.5%) in the infection medium. Untreated samples and samples treated with water (final 12.5%) or cetylpyridinium chloride (CPC) (final 50 μg/mL), which disrupts the integrity of influenza virus particles [41], were used as negative control or positive control, respectively. After incubation at 37°C for 30 min, the virions were fixed with 1% paraformaldehyde in PBS, washed with NTE buffer [10 mM Tris-HCl (pH 8.0), 150 mM NaCl, 1 mM EDTA], and negatively stained with 2% phosphotungstic acid. Images were obtained using a transmission electron microscope (TEM, HT-7700, Hitachi High-Tech, Tokyo, Japan) operating at 80 kV with an XR81-B CCD camera. For each specimen, around one hundred virus particles were recorded, and the morphological changes were evaluated visually. We counted the number of intact and disrupted virus particles from each image and calculated the percentages of disrupted virus particles.

### Statistical analysis

All results are expressed as mean ± the standard error of the mean (SEM). Comparisons among more than two groups were performed using one-way analysis of variance (ANOVA). *p* < 0.05 indicated statistical significance.

## Results

### Cytotoxicity of Y30 extract against MDCK cells

**Fig 1:**
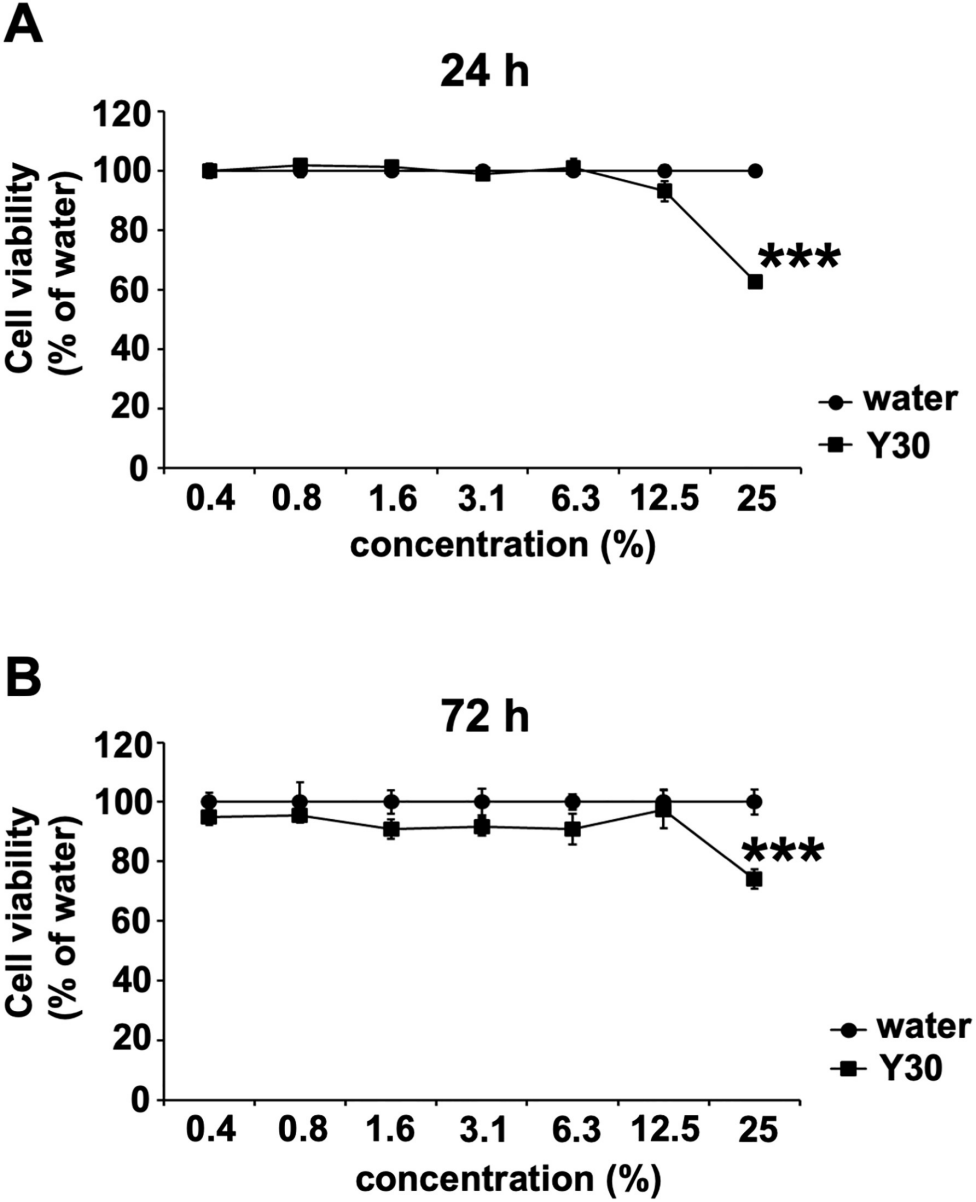
Toxicity of the Y30 extract toward Madin-Darby canine kidney (MDCK) cells. MDCK cells were treated with the Y30 extract (0.4–25%) in water. Cell viability was determined using the thiazolyl blue tetrazolium bromide assay after incubation with the extracts for 24 h (n = 8 each) (A) and 72 h (n = 12 each) (B). Data are expressed as mean ± the standard error of the mean (SEM) of three experiments. ***p < 0.001 relative to water treatment.

To assess the cytotoxicity of the Y30 extract, we performed MTT assay to evaluate the viability of the MDCK cells after incubation for 24 or 72 h in infection medium containing BSA (Fig 1). Compared to MDCK cells exposed to only water, MDCK cells treated with the 25% Y30 extract showed significant reduction in viability after 24 (Fig 1A) and 72 h (Fig 1B) of incubation (*p* < 0.001 each), whereas the 0.4–12.5% Y30 extracts did not affect cell viability. Therefore, the above results suggested that treatment with ≤ 12.5% Y30 extract for 24 and 72 h was not cytotoxic for MDCK cells.

### Y30 extract promoted the survival of influenza A-infected cells and inhibited influenza A virus infection in the co-treatment model

**Fig 2:**
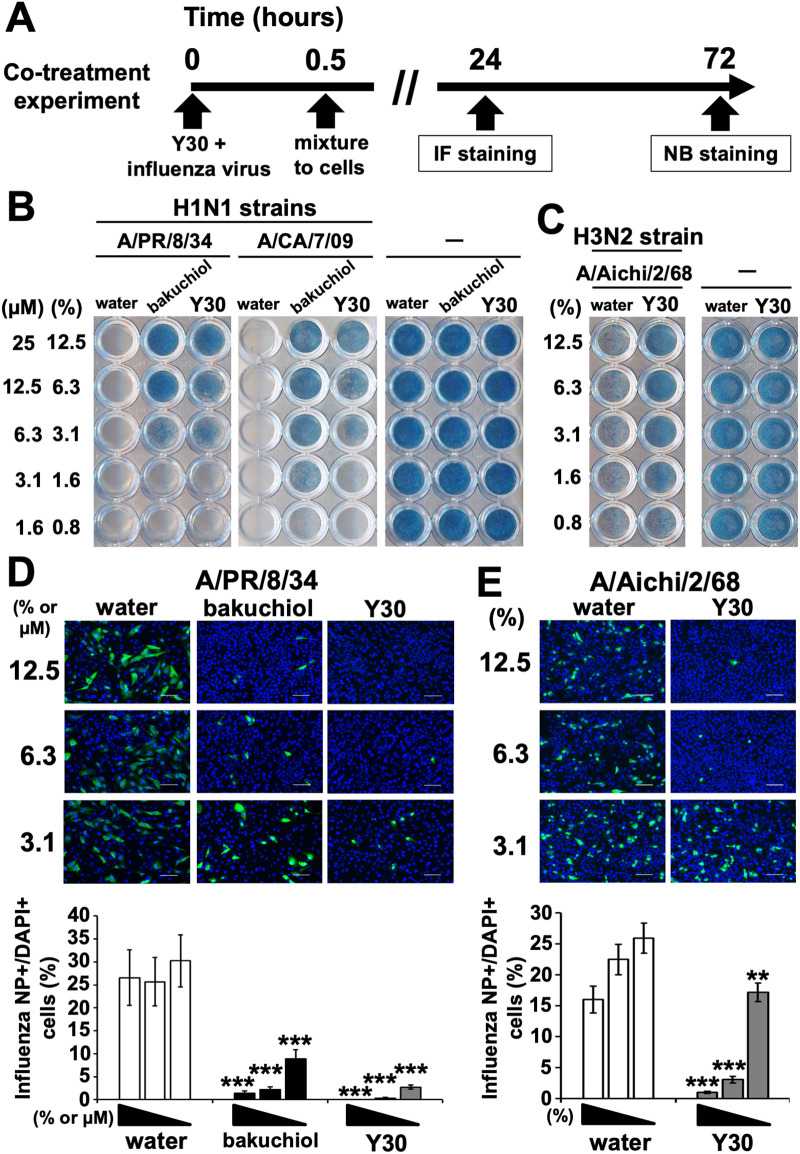
Effects of Y30 extract on the viability of MDCK cells infected with influenza A viruses, and the inhibition of influenza A virus infection in the co-treatment experiment. (A) The scheme of co-treatment experiment which was co-incubated with Y30 extract and influenza A viruses. Immunofluorescence (IF) staining at 24 h and naphthol blue-black (NB) staining at 72 h were performed against the MDCK cells treated with Y30 extract and influenza A viruses. (B and C) The Y30 extracts (final 0.8–12.5%) were mixed with or without the influenza A H1N1 Puerto Rico 8/34 (A/PR/8/34), California 7/09 (A/CA/7/09) (B) or H3N2 Aichi 2/68 (A/Aichi/2/68) (C) viruses [multiplicity of infection (MOI), 10] and subsequently added to MDCK cells. Water (final 0.8–12.5%) and (+)-(*S*)-bakuchiol (bakuchiol, final 1.6–25 μM) were used as negative and positive controls, respectively. After incubation for three days, cell viability was determined via NB staining. Data are representative of three independent experiments. (D and E) The Y30 extracts (final 3.1–12.5%) (n = 9 each) were mixed with A/PR/8/34 (D) or A/Aichi/6/68 (E) viruses at MOI of 0.1, and subsequently added to MDCK cells. Water (final 3.1–12.5%) (n = 9 each) or (+)-(*S*)-bakuchiol (bakuchiol; final 3.1–12.5 μM) (n = 9 each) were used as the negative or positive controls, respectively. Cells were fixed and permeabilized after 24 h of treatment. Infected cells were visualized via IF staining of the influenza A virus nucleoprotein (NP) (green), and the cell nuclei (blue) stained with diamidino-2-phenylindole (DAPI). The cells were subsequently photographed using a fluorescence microscope (D and E upper panels). The percentages of influenza A virus NP-positive cells per DAPI-positive cells were calculated based on the counts of influenza A virus NP-positive and DAPI-positive cells (D and E lower panels). The white scale bar in each image represents 100 μm. Data are expressed as means ± SEM of three independent experiments. **p < 0.01; ***p < 0.001 relative to water treatment.

We evaluated the anti-influenza activity of the Y30 extract in the co-treatment experiment (Fig 2A) to examine whether Y30 extract has a direct virucidal effect or whether it inhibits the entry of influenza A viruses in the host cells.

First, we assessed the survival of MDCK cells after treatment with the Y30 extract and A/PR/8/34, A/CA/7/09 (H1N1pdm09), or A/Aichi/2/68 viruses. Cells exposed to water and infected with each influenza A virus strain were not stained (Fig 2B, left and center panels, and 2C, left panel). Cells treated with the 3.1–12.5% Y30 extract and infected with A/PR/8/34 or A/CA/7/09 viruses (Fig 2B, left and center panels), and also with the 1.6–12.5% Y30 extract and infected with the A/Aichi/2/68 virus (Fig 2C left panel) stained blue, indicating that cells remained viable following exposure to the influenza A H1N1 and H3N2 viruses. Cells treated with 3.1–25 μM bakuchiol showed similar results (Fig 2B, left and center panels). The indicated concentrations of all samples without the addition of viruses stained blue (Fig 2B and 2C, right panels), suggesting that the concentrations used were not cytotoxic to the MDCK cells. Thus, the Y30 extract promoted the survival of MDCK cells infected with influenza A H1N1 and H3N2 viruses. Next, to investigate whether the Y30 extract inhibited influenza A virus infection, we performed NP-immunofluorescence staining of MDCK cells treated with a mixture of the Y30 extract and A/PR/8/34 or A/Aichi/2/68 viruses for 24 h. The cells were observed under a microscope and photographed (Fig 2D and 2E, upper panels). The NP-immunostained cells were counted, and the percentages of NP-positive cells relative to DAPI-positive cells were calculated (Fig 2D and 2E, lower panels). Treatment with the Y30 extract or bakuchiol reduced the number of stained cells (Fig 2D and 2E, upper panels). Treatment with the Y30 extract or bakuchiol significantly reduced the percentages of influenza A NP-positive cells relative to those of the water-treated cells in a concentration-dependent manner (*p* < 0.001 each) (Fig 2D and 2E, lower panels). The above results confirmed the inhibitory effect of the Y30 extract on influenza A virus infection.

Therefore, Y30 extract possesses anti-influenza activity in the co-treatment model, suggesting that Y30 extract has a direct virucidal effect or inhibits the entry of influenza A viruses in the host cells.

### Y30 extract inhibited influenza A virus growth in the pre-infection model

**Fig 3:**
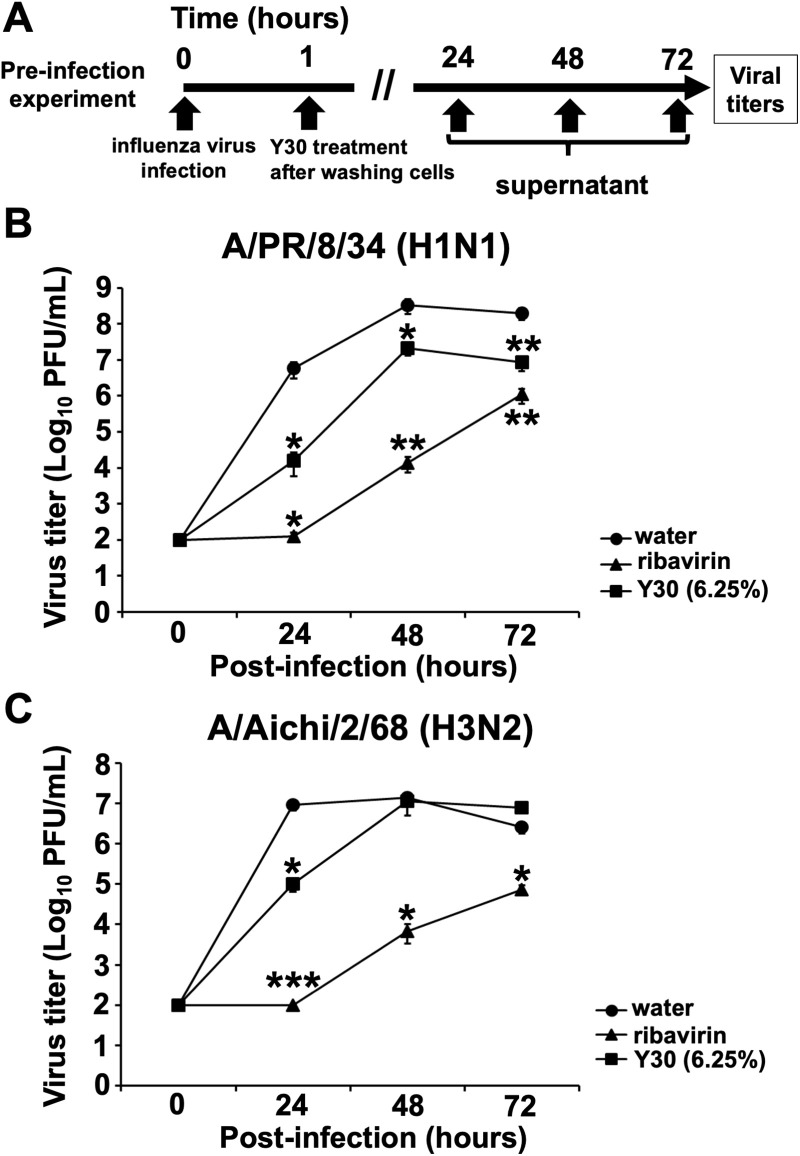
Inhibitory effects of the Y30 extract on influenza A virus growth in the pre-infection experiment. (A) The scheme of pre-infection experiment which involved the treatment with Y30 extract in the influenza A virus-infected MDCK cells. The MDCK cells were infected with A/PR/8/34 (B) or A/Aichi/2/68 (C) viruses at a MOI of 0.001 for 1 h, and the infected cells were washed. The Y30 extract (final 6.25%) (n = 11) was added to the cells in infection medium supplemented with l-tosylamido-2-phenyl ethyl chloromethyl ketone (TPCK)-treated trypsin. Water (final 6.25%) (n = 11) or ribavirin (final 25 μM) (n = 11) were used as the negative or positive controls, respectively. The conditioned culture medium was collected at the indicated time points and subsequently added to MDCK cells. Viral titers [plaque forming units per mL (PFU/mL)] were calculated based on the number of influenza A virus NP-immunostained cells. Data are expressed as mean ± SEM of four independent experiments. *p < 0.05; **p < 0.01; ***p < 0.001 relative to water treatment.

We further evaluated the inhibitory effect of Y30 extract on influenza A virus growth in virus-infected host cells in the pre-infection experiment (Fig 3A) to examine whether Y30 extract inhibits the influenza A virus replication and/or release by targeting viral or host cell factors. In this experiment, we measured viral titers in the cell-condition media following treatment of A/PR/8/34 and A/Aichi/2/68 viruses-infected MDCK cells with the Y30 extract for 24–72 h. Treatment with conditioned media from samples exposed to the Y30 extract significantly reduced the titers of the A/PR/8/34 virus from 24 (*p* < 0.05), 48 (*p* < 0.05), or 72 (*p* < 0.01) h (Fig 3B) and that of the A/Aichi/2/68 virus at 24 h (*p* < 0.05) (Fig 3C) compared to those treated with media from water-treated cells (Fig 3B and 3C). Treatment with conditioned media from samples exposed to ribavirin was used as the positive control, which significantly reduced titers of A/PR/8/34 at 24 (*p* < 0.05), 48 (*p* < 0.01), or 72 (*p* < 0.01) h (Fig 3A) and A/Aichi/2/68 viruses at 24 (*p* < 0.001), 48 (*p* < 0.05), or 72 (*p* < 0.05) h (Fig 3B). The above findings showed that the Y30 extract inhibited the growth of the influenza A virus.

Therefore, the above results demonstrated that the Y30 extract inhibited influenza A H1N1 and H3N2 virus growth in virus-infected host cells, suggesting that this extract inhibits the influenza A virus replication and/or release by targeting viral or host cell factors.

### Y30 extract did not induce anti-influenza activity in the pre-treated cells

**Fig 4:**
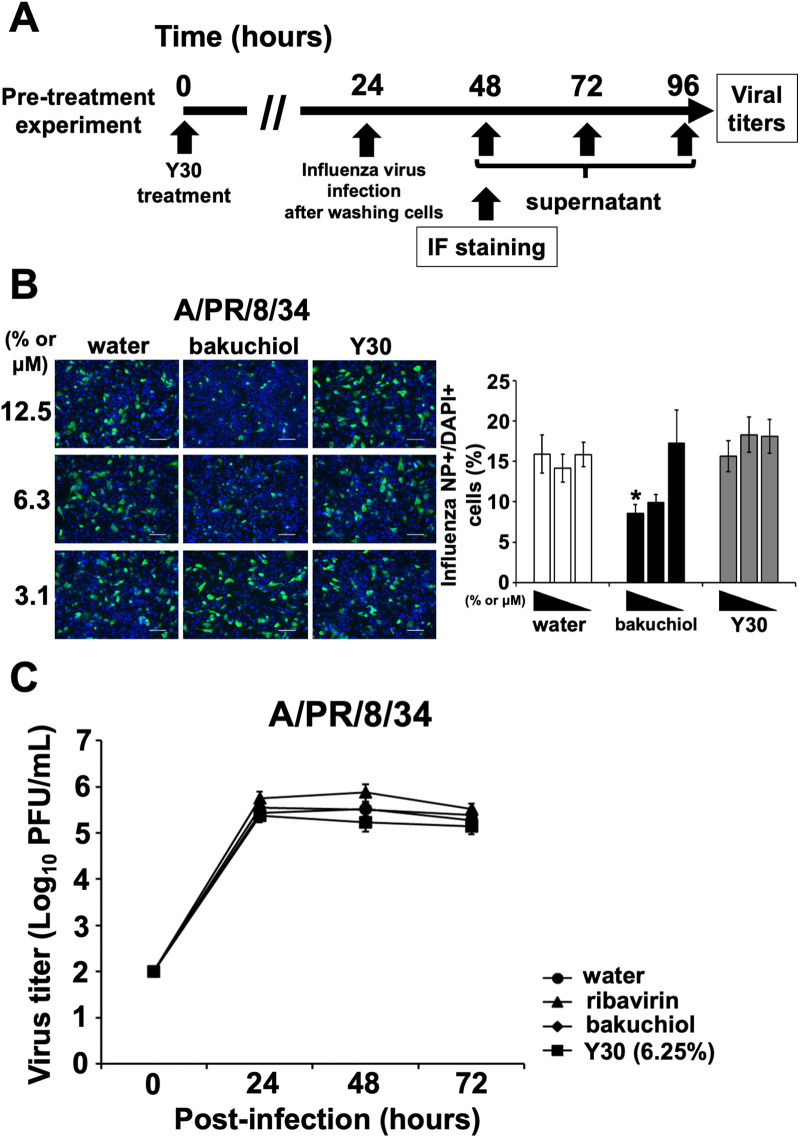
Y30 extract did not inhibit influenza A virus infection and growth in the pre-treatment experiment. (A) The scheme of pre-treatment experiment where Y30 extract-treated MDCK cells were infected with influenza A virus. (B) The MDCK cells were treated with Y30 extract (final 3.1–12.5%) (n = 8 each) for 24 h, and subsequently infected with A/PR/8/34 virus at a MOI of 0.1 after washing the cells. Water (final 3.1–12.5%) (n = 8 each) or (+)-(*S*)-bakuchiol (bakuchiol; final 3.1–12.5 μM) (n = 8 each) were used as the negative or positive controls, respectively. Cells were fixed and permeabilized after 24 h of infection. Infected cells were visualized via immunofluorescence (IF) staining of the influenza A virus NP (green), and the cell nuclei (blue) with DAPI. The cells were subsequently photographed using a fluorescence microscope (B left panel). The percentages of influenza A virus NP-positive cells per DAPI-positive cells were calculated based on the counts of influenza A virus NP-positive and DAPI-positive cells (B right panel). The white scale bar in each image represents 100 μm. Data are expressed as means ± SEM of two independent experiments. *p < 0.05 relative to water treatment. (C) The MDCK cells were treated with Y30 extract (final 6.25%) (n = 15) for 24 h, and subsequently infected with A/PR/8/34 virus at an MOI of 0.1 for 1 h after washing the cells. The infected cells were washed and subsequently added to the cells in infection medium supplemented with TPCK-treated trypsin. Water (final 6.25%) (n = 15) and ribavirin (final 25 μM) (n = 15) or bakuchiol (final 12.5 μM) (n = 15) were used as the negative controls in this experiment. The conditioned culture medium was collected at the indicated time points and subsequently added to MDCK cells. Viral titers (PFU/mL) were calculated based on the number of influenza A virus NP-immunostained cells. Data are expressed as means ± SEM of five independent experiments. **p < 0.01; ***p < 0.001 relative to water treatment, calculated using Student’s *t*-test analysis.

In addition to the co-treatment and pre-infection experiments (Figs 2A and 3A), we performed the pre-treatment experiment (Fig 4A) of host cells pre-treated with Y30 extract to evaluate whether Y30 extract has a direct effect on the host cells, inhibiting the entry, replication, assembly, and/or release of influenza A virus.

First, to investigate whether the Y30 extract inhibited influenza A virus infection in the pre-treatment experiment, we performed NP-IF staining of MDCK cells infected with A/PR/8/34 virus after the treatment with Y30 extract for 24 h. The cells were observed under a microscope and photographed (Fig 4B, left panel). The NP-immunostained cells were counted, and the percentages of NP-positive cells relative to DAPI-positive cells were calculated (Fig 4B, right panel). Treatment with 12.5 μM bakuchiol, which activates nuclear factor erythroid 2-related factor pathway in host cells and induces anti-influenza effect [34], reduced the number of stained cells and the percentages of influenza A NP-positive cells significantly relative to those of the water-treated cells (*p* < 0.05) (Fig 4B); Y30 extract treatment did not cause such reduction (Fig 4B). These results indicated that the cells pre-treated with Y30 extract do not inhibit influenza A virus infection. Next, to evaluate the inhibition of the influenza A virus growth in host cells pre-treated with Y30, we measured viral titers in the cell-condition media following the infection of Y30 extract-pre-treated MDCK cells with A/PR/8/34 virus for 24–72 h. Treatment with conditioned media from samples exposed to the Y30 extract did not reduce the titers of the A/PR/8/34 virus between 24–72 h compared to treatment with media from water-treated cells. Samples exposed to ribavirin, the target of the viral RdRp, and bakuchiol were not reduced (Fig 4C). The above results indicate that cells pre-treated with Y30 extract did not inhibit influenza A virus growth.

Taken together, the above results demonstrated that cells pre-treated with Y30 extract did not inhibit influenza A H1N1 virus infection and growth, suggesting that this extract has a direct effect on influenza viruses or host factors that inhibit the replication, assembly, and/or release of influenza A virus when used against influenza viruses or to treat influenza viruses-infected host cells.

### Y30 extract did not inhibit influenza A virus HA, NA, and RNA polymerase activities

While co-incubating the extracts and influenza virus in advance for the virus-infected cell viability and infection experiments (Fig 2), the Y30 extract may inhibit influenza A viral binding to host cells by targeting viral hemagglutinin. Infection with the influenza virus is initiated by the binding of the viral hemagglutinin surface protein to sialic acid residues present in the glycoproteins and glycolipids on the host cell surface [42]. HA of erythrocytes occurs when viral hemagglutinin binds to sialic acid on the erythrocyte surface.

**Fig 5:**
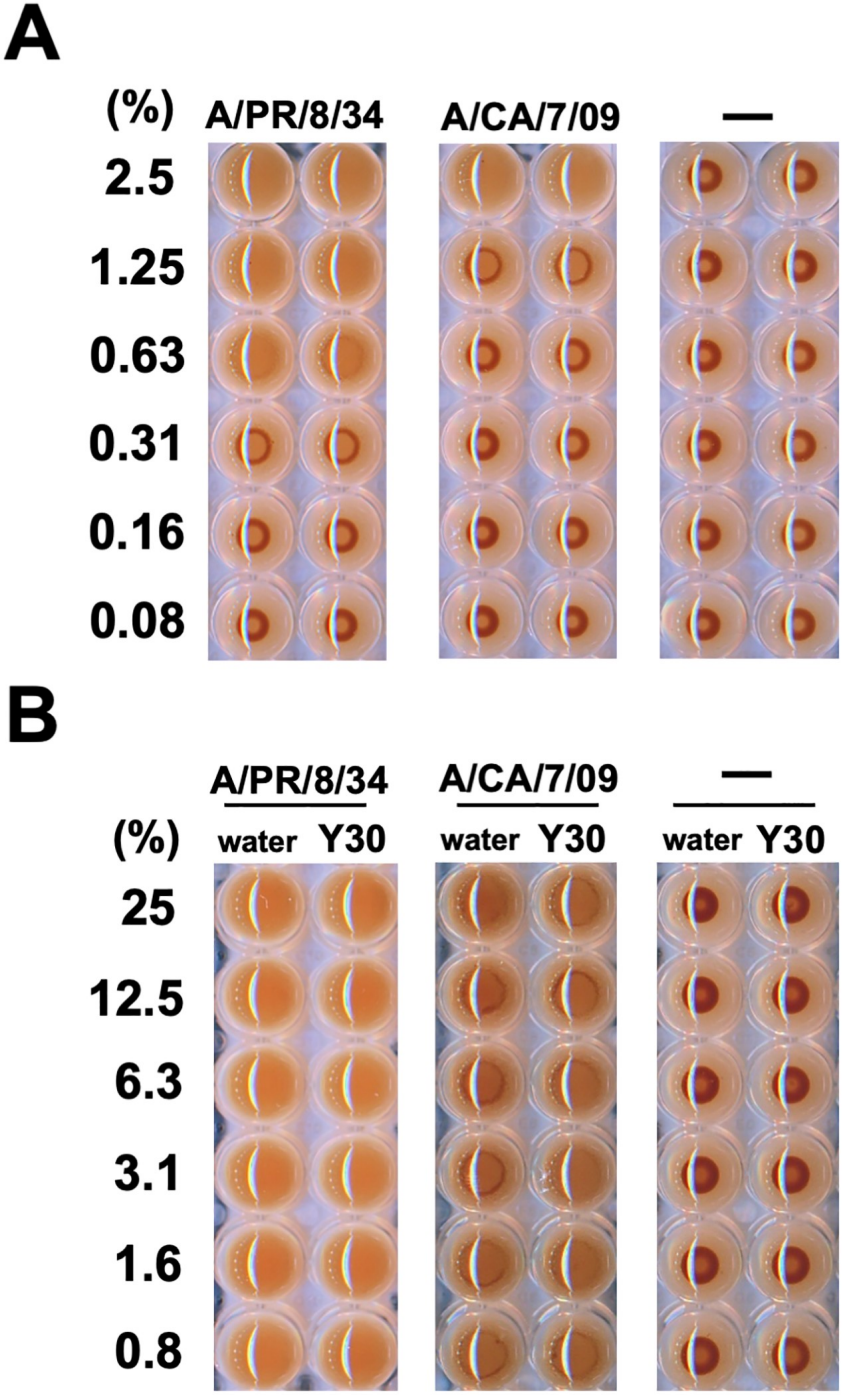
Y30 extract did not inhibit the hemagglutination (HA) activities of influenza A virus. (A) The HA activities of the A/PR/8/34 and A/CA/7/09 virus particles. The viruses were serially diluted with phosphate-buffered saline (PBS) to obtain the indicated percentages and subsequently added to the rabbit red blood cells (rRBCs). Plates were photographed after 1 h of incubation. (B) The inhibitory effect of the Y30 extract on the HA activities of influenza A H1N1 virus. The Y30 extract was diluted (final 0.8–25%) with PBS and subsequently infected with or without 1.25% A/PR/8/34 or 2.5% A/CA/7/09 viruses. Water (final 0.8–25%) was used as the negative control. Each sample was mixed with 0.5% rRBCs. Plates were photographed after 1 h of incubation. Data are representative of three independent experiments.

As mentioned above, we showed that the Y30 extract inhibited influenza A virus infection in the co-treatment experiment (Fig 2). Hence, we investigated the effect of the Y30 extract on HA of erythrocytes by influenza A viral hemagglutinin. Compared to the no virus control, rRBCs were aggregated by 0.63% or 2.5% A/PR/8/34 or A/CA/7/09 viruses, respectively (Fig 5A, left and center panels). The Y30 extract did not inhibit the HAI activities of the A/PR/8/34 and A/CA/7/09 viruses compared to that of the water control (Fig 5B, left and center panels). Thus, these results suggested that the Y30 extract-mediated inhibition of virus infection does not occur via inhibition of the HA activity of the influenza A virus.

**Fig 6:**
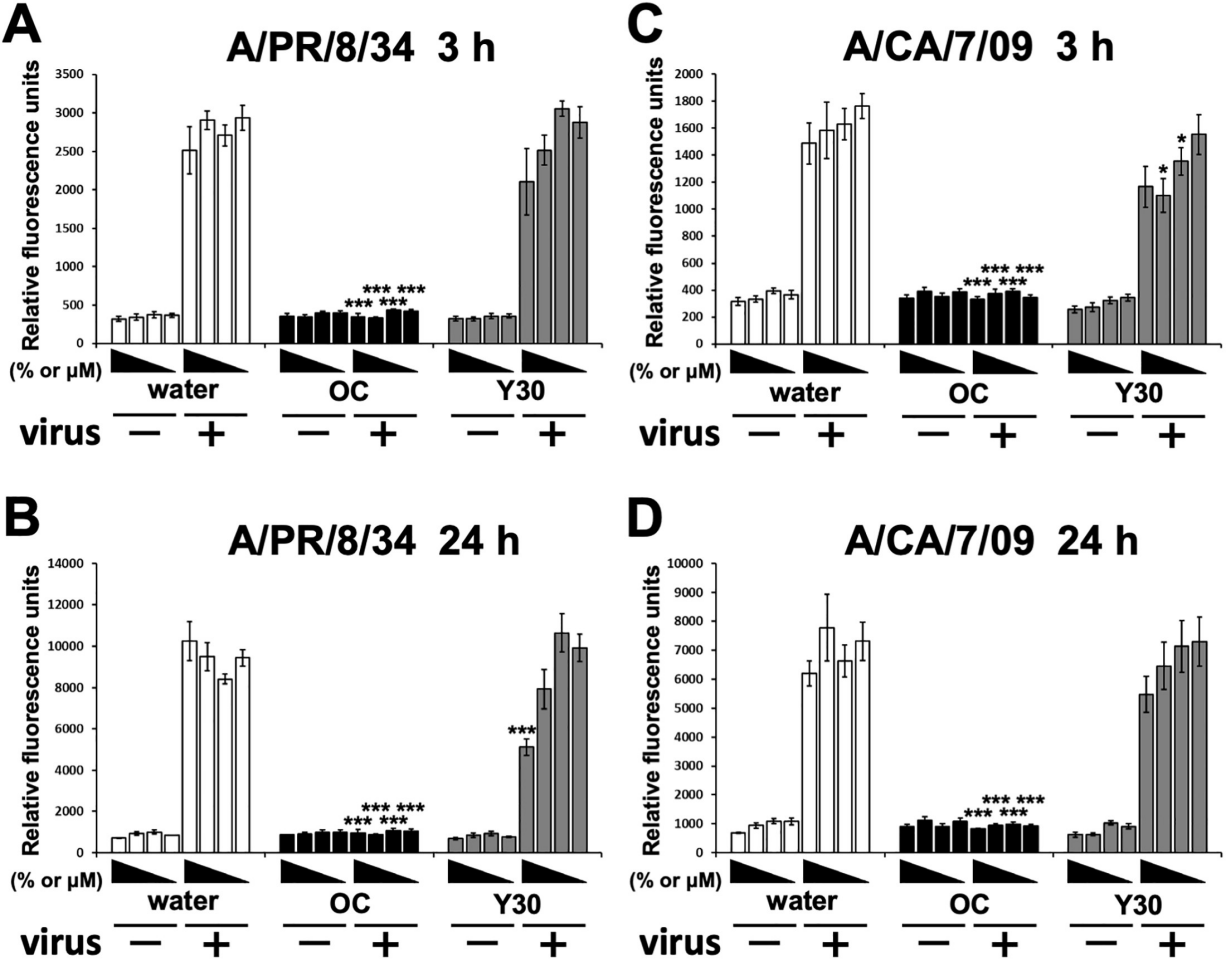
Inhibitory effects of the Y30 extract on the NA activities of influenza A viruses. The Y30 extract (final 3.1–25%) was diluted in a 96-well black plate. Water (final 3.1–25%) or oseltamivir carboxylate (OC) (final 3.1–25 μM) were used as the negative or positive controls, respectively. Each well was treated with or without A/PR/8/34 or A/CA/7/09 viruses. Each sample was mixed with 2ʹ-(4-methylumbelliferyl)-α-D-*N*-acetylneuraminic acid. The relative fluorescence units in A/PR/8/34 virus-treated wells at 3 h (A) or 24 h (B) (n = 6 each) or A/CA/7/09 virus-treated wells at 3 h (C) or 24 h (D) (n = 6 each) were measured at excitation wavelength of 365 nm and emission wavelength of 445 nm. Data are expressed as mean ± SEM of three independent experiments. *p < 0.05; ***p < 0.001 relative to water treatment.

Fig 3 shows that treatment with the Y30 extract reduced the viral titers released from A/PR/8/34 and A/Aichi/2/68 viruses-adsorbed MDCK cells. This may indicate that the Y30 extract inhibited the release of virions from the membrane of the host cell via the sialidase activity of viral NA and/or that the viral genome replicated in the host nucleus using viral RdRp [42]. First, to examine whether each Y30 extract inhibited the sialidase activity of the influenza virus NA, we performed the NA assay with influenza A H1N1 viral particles (A/PR/8/34 and A/CA/7/09 viruses) (Fig 6). Compared to that of water, the Y30 extract (25%) inhibited the sialidase activity of the A/PR/8/34 virus particle after 24 h incubation (*p* < 0.001) (Fig 6B), while the 6.25–12.5% Y30 extract inhibited the sialidase activity of A/CA/7/09 virus particles after 3 h (*p* < 0.05) (Fig 6C). Compared to that of water, oseltamivir carboxylate, a NA inhibitor, strongly inhibited the sialidase activity of the A/PR/8/34 and A/CA/7/09 virus particles after 3 h and 24 h in a concentration-dependent manner (*p* < 0.001 each) (Fig 6).

**Fig 7:**
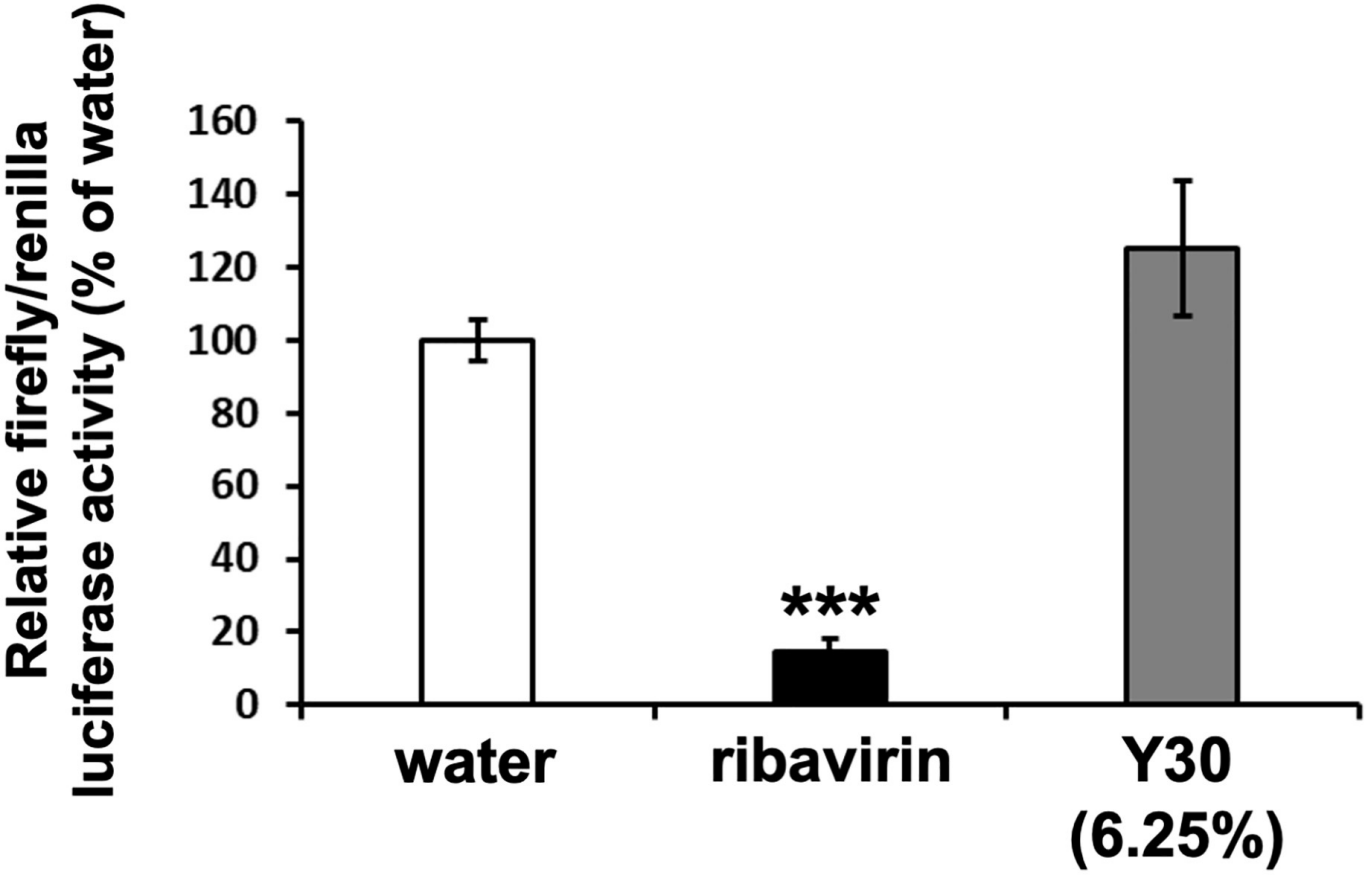
Y30 extract did not inhibit the RNA-dependent RNA polymerase (RdRp) activity of influenza A virus. The inhibitory effects of the Y30 extract on influenza A virus RdRp activity were evaluated using the influenza A virus minigenome assay based on the dual luciferase system in MDCK cells. Firefly and *Renilla* luciferase activities were measured in the lysates of transfected cells treated with the Y30 extract (final 6.25%), water (final 6.25%) or ribavirin (final 25 μM) (n = 9 each). Luciferase activity was expressed relative to that of the water-treated cells (set as 100%). Data are presented as the mean ± SEM of three independent experiments. ***p < 0.001 relative to water treatment.

Next, to determine whether each Y30 extract inhibited the RdRp activity of the influenza A virus, we performed the minigenome assay based on the dual luciferase system in RdRp-expressing MDCK cells. Compared to the water control, treatment with the 6.25% Y30 extract did not inhibit influenza A H1N1 viral RdRp activity in MDCK cells (Fig 7), while ribavirin, an inhibitor of viral genome replication, significantly inhibited viral RdRp activity (*p* < 0.001) (Fig 7). Thus, these results showed that the Y30 extract did not inhibit the RdRp activity of the influenza A H1N1 virus. We have previously shown that the 6.25% Y30 extract inhibited A/PR/8/34 virus growth (Fig 4A). Therefore, the Y30 extract-mediated inhibition of influenza A virus growth does not occur via inhibition of the viral NA and RdRp activities.

Taken together, these results suggested that the Y30 extract contains inhibitory compounds that target other viral or host cell factors required for influenza A virus infection and growth.

### Y30 extract disrupted the integrity of influenza A virus particles

The above results where Y30 inhibits influenza A virus infection (Fig 2) but does not result in the hemagglutinin-induced viral binding in the co-treatment experiment (Fig 5) suggests that Y30 extract has a direct virucidal effect, such as damaging the integrity of influenza A virus particles. Thus, to evaluate the disruptive effect of Y30 extract against the integrity of influenza A virus particles, we analyzed influenza A virus particles which were treated with Y30 extract in a cell-free co-treatment experiment using a TEM.

**Fig 8:**
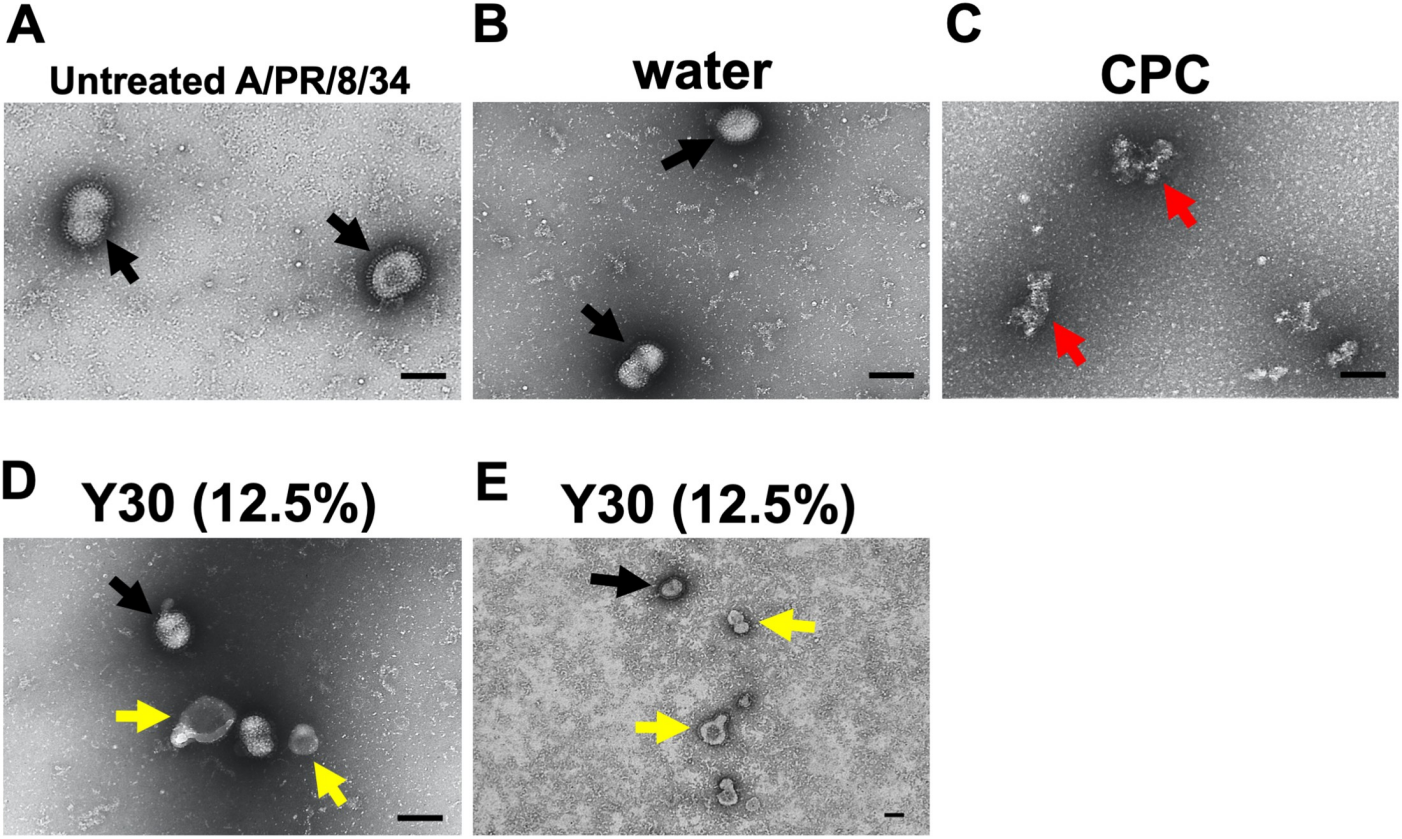
Disruptive effect of Y30 extract on the integrity of influenza A virus particles. The supernatant of MDCK cells infected with A/PR/8/34 virus was mixed with untreated (A), and water (final 12.5%) (B), cetylpyridinium chloride (CPC) (final 50 μg/mL) (C), or Y30 extract (final 12.5%) (D and E) -treated samples in the infection medium. After incubation for 30 min, the fixed-virions were negatively stained, and the images were obtained using a transmission electron microscope. The morphological changes were visually evaluated from each specimen, and intact (black arrows) and disrupted (red and yellow arrows) virus particles were observed. Then, these viruses were counted, and the percentages of disrupted virus particles were calculated. The black scale bar in each image represents 100 nm. We found that 31.6% (31/98) or 93.7% (119/127) of the viruses treated with Y30 extract or CPC were disrupted, respectively, whereas 16.2% (17/105) of the viruses treated with water were disrupted. In untreated viruses, only 6.5% (6/93) were disrupted. Data are representative of two independent experiments, and these results were reproducible.

TEM analysis showed that the viral envelope of untreated virus (Fig 8A) or water-treated A/PR/8/34 virus (Fig 8B) were intact (Fig 8, black arrows). However, the exposure to Y30 extract (Fig 8D and 8E) induced the disruption of A/PR/8/34 viral envelope, and the part of viral envelope found to be missing suggested the permeabilization of viral membrane envelope (Fig 8D and 8E, yellow arrows). The exposure to CPC (Fig 8C) strongly induced the disruption of A/PR/8/34 viral envelope, such that the original form of virus particles could not be recognized (Fig 8C, red arrows), as reported previously [41]. We quantified the percentages of intact and disrupted virus particle after each sample treatment and found that 31.6% (31/98) or 93.7% (119/127) of the viruses treated with Y30 extract or CPC were disrupted, respectively, whereas only 6.5% (6/93) or 16.2% (17/105) of viruses untreated or treated with water were disrupted, respectively. These data revealed that Y30 extract disrupted the integrity of A/PR/8/34 virus particles.

Therefore, these results demonstrated that Y30 extract has a direct virucidal effect on the disruption of integrity of the influenza A virus particles by permeabilizing the viral membrane envelope.

### Water or ethyl acetate Y30 extracts promoted the survival of influenza A-infected cells

To identify the anti-influenza compounds present in the Y30 extract, we prepared water and EtOAc extracts from the Y30 extract. We evaluated the anti-influenza A activity of the water and EtOAc extracts by assessing the survival of MDCK cells after treatment with each extract and A/PR/8/34 virus. Cells exposed to water and infected with A/PR/8/34 virus did not stain (Fig 8, left panel), whereas cells treated with 0.4–12.5% water or 3.1–12.5% EtOAc extract and infected with A/PR/8/34 virus stained blue (Fig 8, left panel). Cells treated with the 1.6–12.5% Y30 extract or 0.8–12.5% Y30 extract of different lots showed similar results (Fig 8, left panel), indicating that the cells remained viable following exposure to the A/PR/8/34 virus. The indicated concentrations of all samples without the addition of viruses stained blue (Fig 8, right panel). Therefore, compared to the EtOAc extract, the water extract of Y30 extract highly promoted the survival of MDCK cells infected with the A/PR/8/34 virus at lower concentration. These results suggested that more anti-influenza compounds were present in the water extract of Y30 extract than in the EtOAc extract.

## Discussion

**Fig 9:**
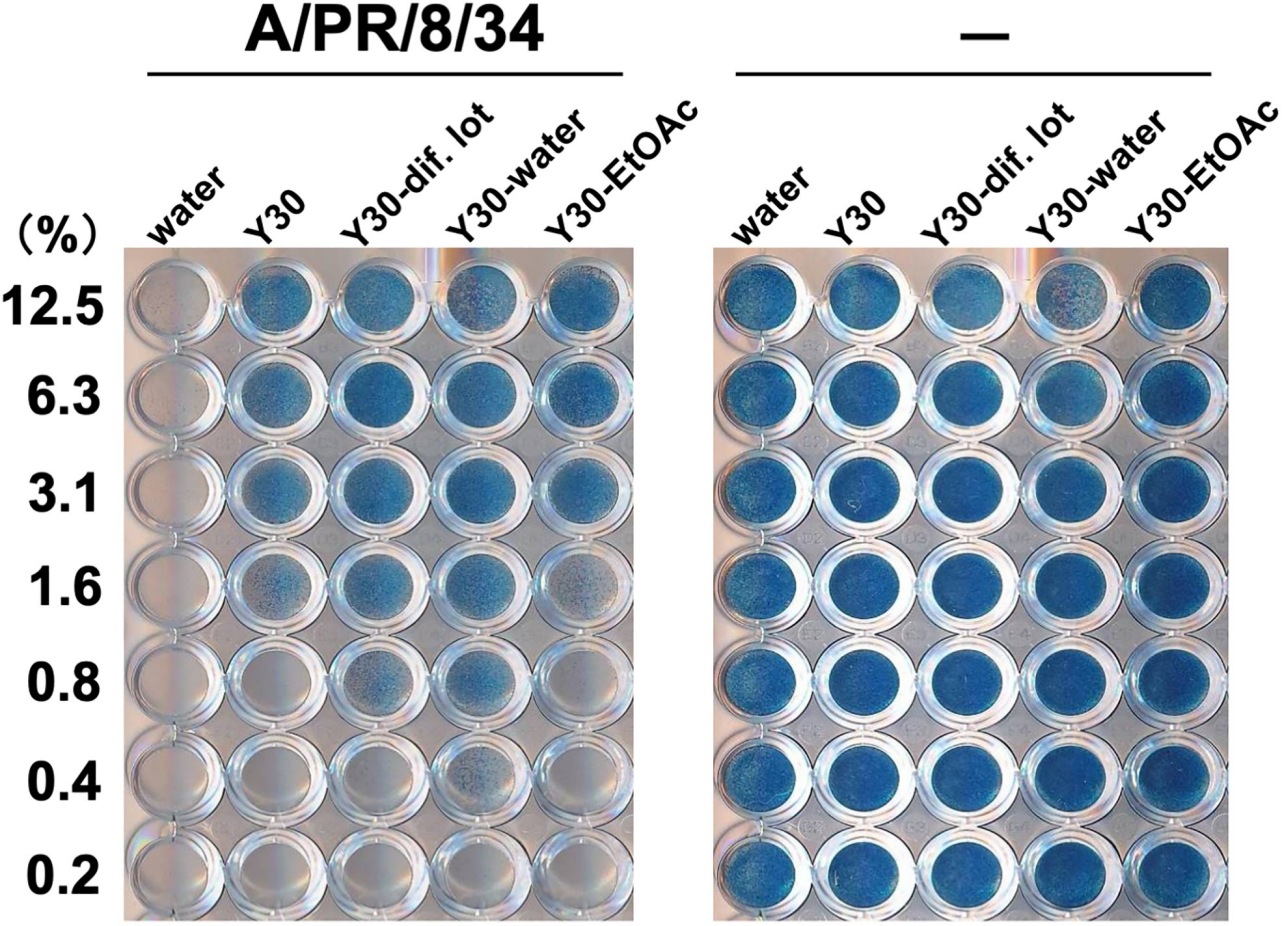
Effect of water or ethyl acetate extracts from the Y30 extract on the viability of MDCK cells infected with the influenza A virus. The inhibitory effects of the water or ethyl acetate (EtOAc) extracts of the Y30 extract on influenza A H1N1 virus infection were evaluated. The Y30 extract, Y30 extract of different lots (Y30-dif. lot), and water (Y30-water) or EtOAc (Y30-EtOAc) extracts from the Y30 extract (final 0.8–12.5% each) were mixed with or without the A/PR/8/34 virus (MOI; 10) and subsequently added to MDCK cells. Water (final 0.2–12.5%) was used as the negative control. After incubation for three days, cell viability was determined via naphthol blue-black staining. Data are representative of three independent experiments.

In the present study, we demonstrated that the Y30 extract strongly promoted the survival of A/PR/8/34, A/CA/7/09, or A/Aichi/2/68 viruses-infected MDCK cells and inhibited A/PR/8/34 or A/Aichi/2/68 viruses infection (Fig 2) and growth (Fig 3) in the co-treatment and pre-infection experiment, respectively. In addition to these two experiments, the pre-treatment experiment revealed that Y30 inhibits influenza A virus infection and growth (Fig 4), suggesting that Y30 extract has a direct effect on influenza viruses or host factors that inhibit the replication, assembly, and/or release of influenza A virus when used against influenza viruses or to treat influenza viruses-infected host cells. To explain the anti-influenza A virus infection and growth properties of the Y30 extract by targeting viral factors, we assessed its effects on the HA, NA, and RNA polymerase activities of the virus (Figs 5–7). However, the Y30 extract did not significantly affect influenza A virus HA, NA, and RdRp activities, indicating that anti-influenza compounds present in the Y30 extract inhibit influenza A virus infection and growth by targeting other viral or host factors for viral assembly and/or release in host cells. Based on the results of Y30 extract inhibiting influenza A virus infection, but not supporting the hemagglutinin-induced viral binding in the co-treatment experiment, we inferred a direct virucidal effect of Y30 extract. TEM analysis revealed that Y30 extract has a disruptive effect against the integrity of influenza A virus particles by permeabilizing the viral membrane envelope (Fig 8). Furthermore, we observed that compared to the EtOAc extract, the water extract of Y30 extract considerably promoted the survival of cells infected with the A/PR/8/34 virus (Fig 9). These results suggested that more anti-influenza components were present in the water extract of Y30 extract than in the EtOAc extract. Our results highlighted the potential of an aqueous rice extract fermented with *A*. *oryzae* and *S*. *cerevisiae* as an anti-influenza medicine and drug source for the development of anti-influenza compounds.

To reveal the mechanism of anti-influenza activity of Y30 extract, we performed three experiments, viz., the co-treatment (Fig 2A), pre-infection (Fig 3A), and pre-treatment (Fig 4A) experiments, having different durations of exposure to Y30 extract for influenza viruses or host cells. In addition, we assessed its effects on the HA, NA, and RNA polymerase activities of the virus (Figs 5–7). These results demonstrated that the Y30 extract inhibits influenza A virus infection and growth, deterring influenza A virus assembly and/or release, but not infection, in host cells. Systematic analysis by Watanabe et al. [43] identified nine host factors responsible for viral replication, viz., Bcl2-associated athanogene 3, bromodomain-containing protein 8, coiled-coli domain-containing protein 135, DEAD-box helicase 55, dolichyl-phosphate mannosyltransferase subunit 3, eukaryotic elongation factor 2, insulin-like growth factor 2 mRNA-binding protein 2, keratin-14, and S100 calcium-binding protein A4. These proteins are known to play important roles during the viral life cycle, such as binding, internalization, and/or translocation of viral ribonucleoprotein complexes into the nucleus by binding influenza viral proteins, such as HA, NA, M, non-structural protein, or RdRp proteins. Therefore, Y30 extract may contain inhibitory compounds against host factors that are required during the influenza life cycle.

In addition, in the co-treatment experiment and TEM analysis (Figs 2 and 8), we demonstrated that Y30 extract inhibits the influenza A virus infection by a direct virucidal effect by disrupting the integrity of influenza A virus particles via permeabilization of the viral membrane envelope. Antiviral agents that disrupt the viral envelope show broad antiviral effect, and are difficult to induce resistance because the viral envelope is derived from the host cell membrane when its particle is formed, which the utilization of host cell membrane cannot be controlled by virus [41,44–50]. Enveloped viruses, and not naked viruses, are sensitive to chemicals [41,50,51], peptides [45,48,49], and lipoproteins [46,52] which induce the degeneration, permeabilization, or perforation of viral membrane. However, these agents have a cytotoxic activity because the disruption of membrane integrity is not only induced for virus but also for the host cell. We found that treatment with a high concentration (25%) of Y30 extract was toxic for MDCK cells. Conversely, the treatment with less than 12.5% of Y30 extract induced anti-influenza activity and disruption of the integrity of influenza A virus particles without the induction of cytotoxicity. Therefore, Y30 extract may contain the agents that disrupt the integrity of influenza A viral particles by degenerating, permeabilizing, or perforating the viral membrane.

Rice Power^®^ extracts without ethanol are produced using techniques similar to that used for producing Japanese rice wine, “sake”. Sake is made with *koji*, *moto*, steamed rice, and water. *Koji* is produced with *A*. *oryzae*, which converts rice starch to sugar, and *moto* is produced with *koji* and *S*. *cerevisiae*, which converts sugar to ethanol [53,54]. Several chemical components and metabolites are produced during these fermentative processes. The chemical components and metabolites generated from *A*. *oryzae* or *S*. *cerevisiae* are related to the flavor, taste, and quality of sake [53]. Akaike et al. showed that twenty-four chemical components are present in twenty different sakes, but did not identify them [53]. Kiyono et al. identified 19 pyroglutamyl peptides in sake, and pyroGlu-Gln and pyroGlu-Leu were the major components [55]. PyroGlu-Leu has been reported to possess anti-microbial [56] and anti-inflammatory [57] activities. Tatsukami et al. identified four peptides, [Leu/Ile]-[Leu/Ile]-[Leu/Ile], Phe-Pro-[Leu/Ile], [Leu/Ile]-[Leu/Ile]-[Leu/Ile]-Pro, and [Leu/Ile]-[Leu/Ile]-[Leu/Ile]-[Leu/Ile]-Pro, and predicted two compounds, the steroidal agent androsterone, identified as C_19_H_28_O, and angeloyloxylupanine or acetoxymatrine, identified as C_17_H_26_N_2_O_3_, in the fermentative process of “yamahai-ginjo-shikomi” sake [54]. Thus, characteristic peptides are generated from rice fermented with *A*. *oryzae* and *S*. *cerevisiae* during sake production. Furthermore, Vilas Boas et al. reviewed antiviral peptides from plants, arthropods, marine organisms, amphibians, and mammals against several viruses, such as human immunodeficiency virus, dengue virus, herpes simplex virus, hepatitis C virus, severe acute respiratory syndrome coronavirus, and influenza virus [58]. Anti-influenza virus peptides have been reported to inhibit virus infection and growth by targeting the viral membrane envelope [45,48,49]. In addition, we found that compared to the EtOAc extract, the water extract of Y30 extract considerably promoted the survival of cells infected with the influenza A virus, suggesting that more anti-influenza components were present in the water extract of Y30 extract than in the EtOAc extract, and these components were water soluble. Therefore, Y30 extract may contain the water-soluble anti-influenza viral peptides or compounds that disrupt the integrity of viral membrane envelope but do not inhibit the HA of erythrocytes by influenza virus hemagglutinin. We intend to isolate the anti-influenza components from water or EtOAc extracts of Y30 extract in future.

### Conclusion

In conclusion, we demonstrated for the first time that the Y30 extract strongly promoted the survival of influenza A virus-infected MDCK cells by inhibiting virus infection and growth in the co-treatment and pre-infection experiments, respectively. In addition, the pre-treatment experiment revealed that Y30 extract inhibits influenza A virus growth but not infection, suggesting that the Y30 extract has a direct effect on influenza viruses or host factors that inhibit the replication, assembly, and/or release of influenza A virus when used against influenza viruses or to treat influenza viruses-infected host cells. However, the Y30 extract did not significantly affect viral HA, NA, and RdRp activities, suggesting that the anti-influenza compounds in the Y30 extract inhibit influenza A virus infection and growth by targeting other viral or host factors for viral assembly and/or release in the host cells. Interestingly, we found that Y30 extract has a disruptive effect against the integrity of influenza A virus particles by permeabilizing the viral membrane envelope. Furthermore, we observed that compared to the EtOAc extract, the water extract of Y30 extract highly promoted the survival of cells infected with A/PR/8/34 virus, suggesting that more potent anti-influenza compounds were present in the water extract of Y30 extract than in the EtOAc extract. Therefore, an aqueous rice extract fermented with *A*. *oryzae* and *S*. *cerevisiae* can be used as an anti-influenza medicine and a crude source of compounds for the development of anti-influenza drugs.

## Supporting information

S1 Raw data (XLSX)

S2 Raw data (ZIP)

S3 Raw data (ZIP)

S4 Raw data (ZIP)

S5 Raw data (ZIP)

S6 Raw data (ZIP)

S7 Raw data (ZIP)

S8 Raw data (ZIP)

S9 Raw data (ZIP)

S10 Raw data (ZIP)

S11 Raw data (XLSX)
