# Supplementary figures and images for: A novel aqueous extract from rice fermented with Aspergillus oryzae and Saccharomyces cerevisiae possesses an anti-influenza A virus activity
Source: PLoS One. 2021 Jan 15;16(1):e0244885. doi: 10.1371/journal.pone.0244885 (PMC7810313; doi:10.1371/journal.pone.0244885)

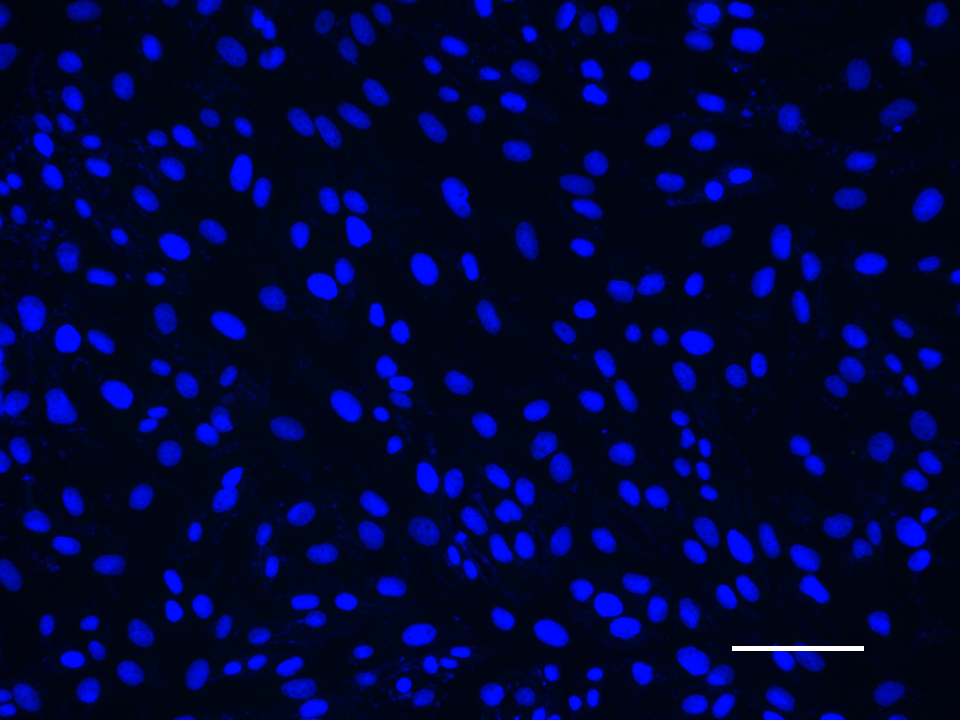

Supplement: S2 Raw data — (ZIP) [file pone.0244885.s002.zip › Y30 Fig. 2/Y30 IFA/Y30 PR8 IFA/170512 Y30 IFA HR-paper Fig/170224 Yusin ekisu Y30 25% #1 overlay Fig-bar.tif]

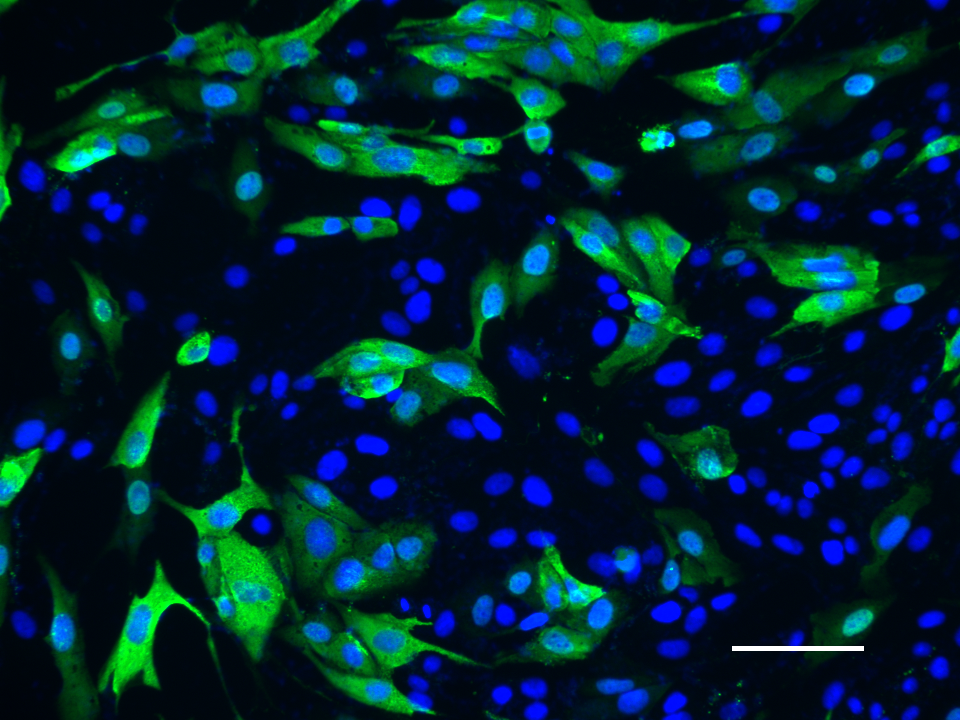

Supplement: S2 Raw data — (ZIP) [file pone.0244885.s002.zip › Y30 Fig. 2/Y30 IFA/Y30 PR8 IFA/170512 Y30 IFA HR-paper Fig/170224 Yusin ekisu H2O 25% #2 overlay Fig-bar.tif]

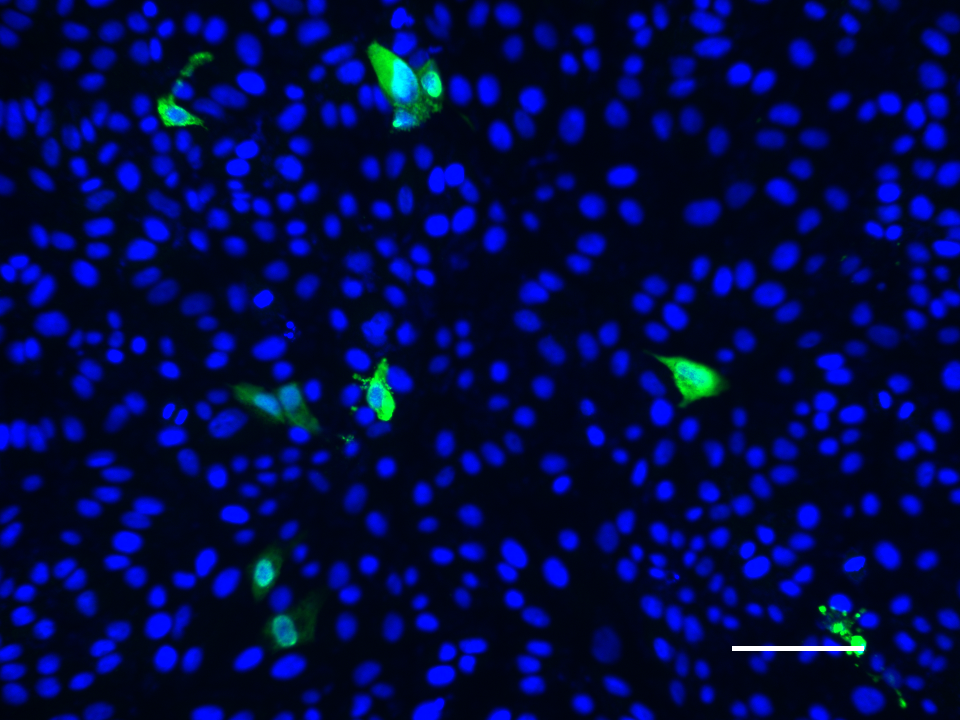

Supplement: S2 Raw data — (ZIP) [file pone.0244885.s002.zip › Y30 Fig. 2/Y30 IFA/Y30 PR8 IFA/170512 Y30 IFA HR-paper Fig/170224 Yusin ekisu bakuchiol 6.25uM #1 overlay Fig-bar.tif]

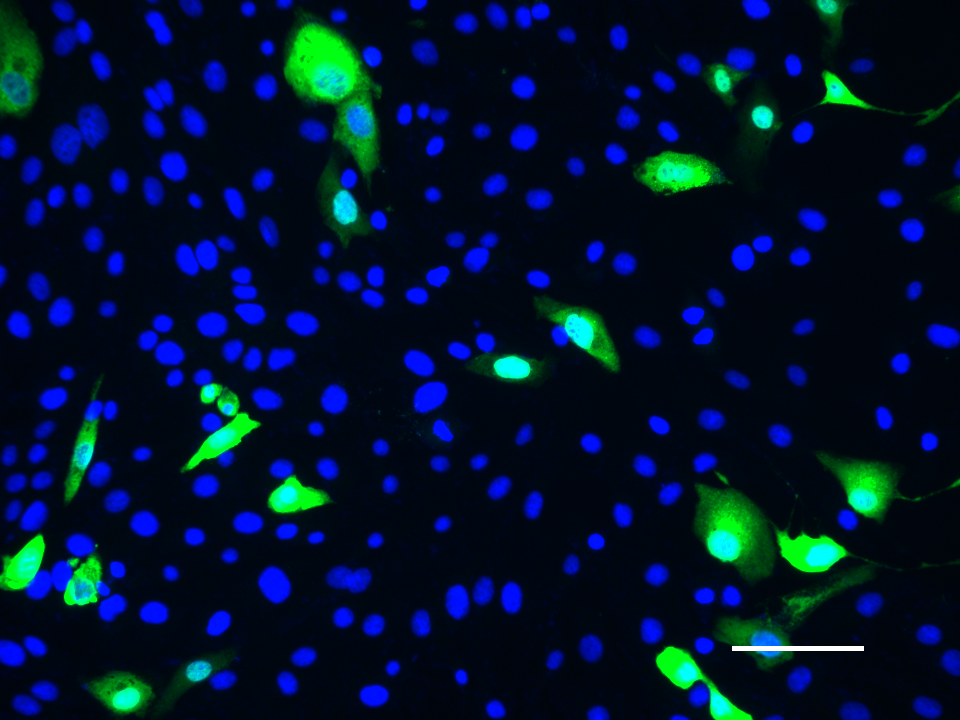

Supplement: S2 Raw data — (ZIP) [file pone.0244885.s002.zip › Y30 Fig. 2/Y30 IFA/Y30 PR8 IFA/170512 Y30 IFA HR-paper Fig/170224 Yusin ekisu bakuchiol 3.125uM #2 overlay Fig-bar.tif]

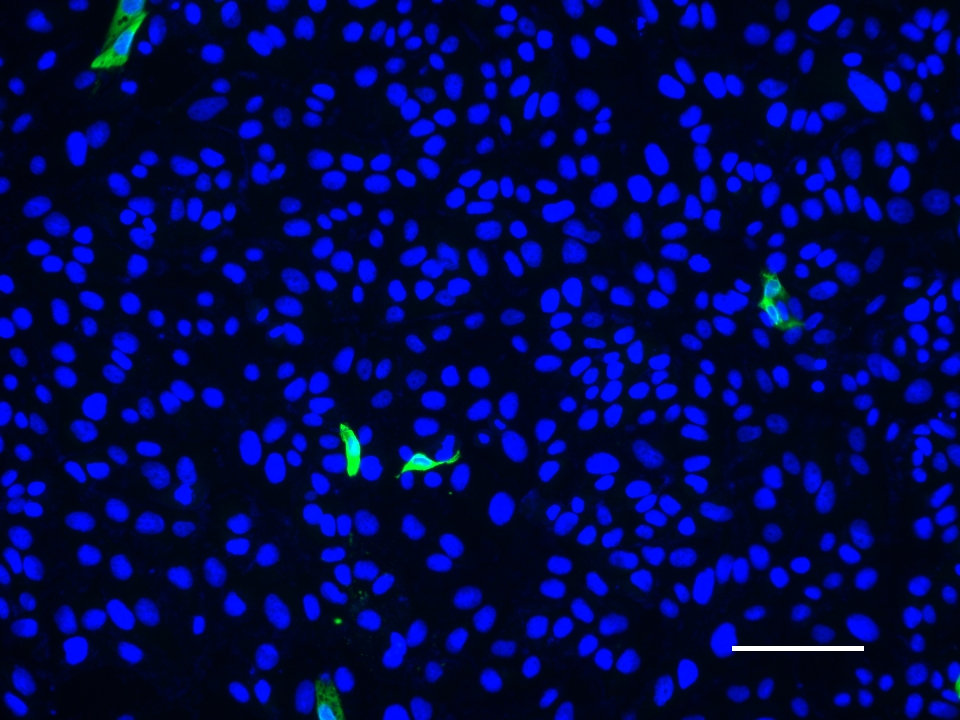

Supplement: S2 Raw data — (ZIP) [file pone.0244885.s002.zip › Y30 Fig. 2/Y30 IFA/Y30 PR8 IFA/170512 Y30 IFA HR-paper Fig/170224 Yusin ekisu bakuchiol 25uM #1 overlay Fig-bar.tif]

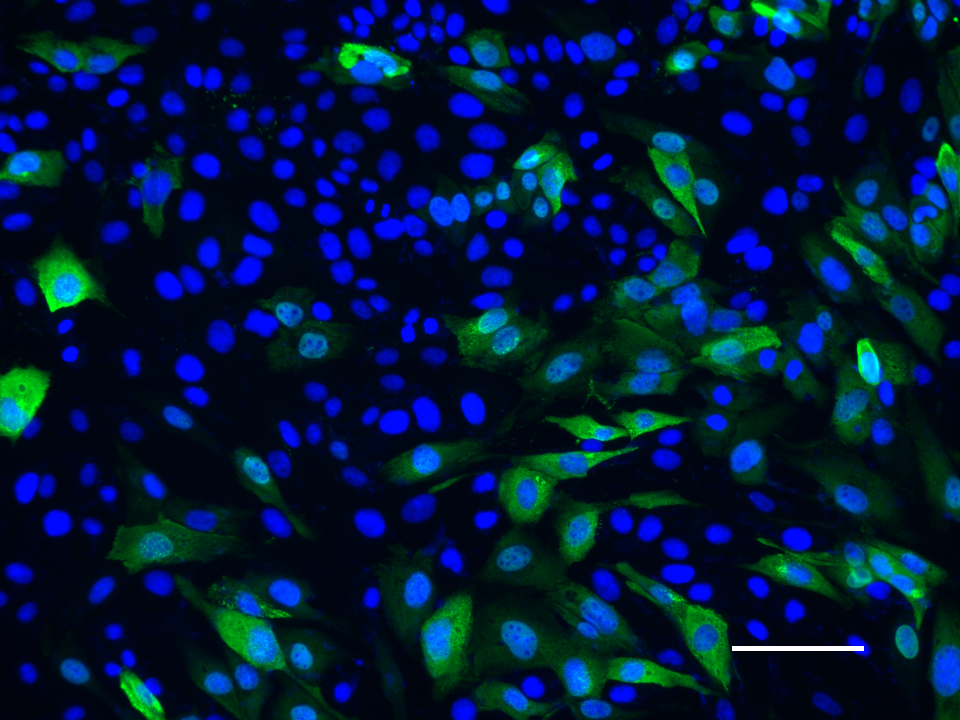

Supplement: S2 Raw data — (ZIP) [file pone.0244885.s002.zip › Y30 Fig. 2/Y30 IFA/Y30 PR8 IFA/170512 Y30 IFA HR-paper Fig/170224 Yusin ekisu H2O 6.25% #1 overlay Fig-bar.tif]

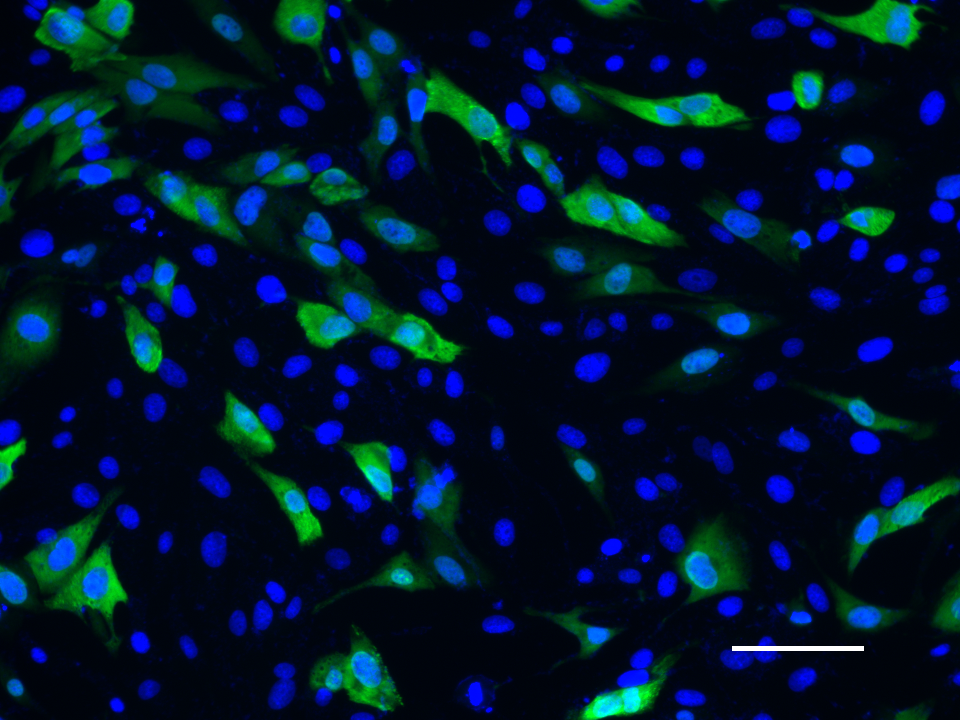

Supplement: S2 Raw data — (ZIP) [file pone.0244885.s002.zip › Y30 Fig. 2/Y30 IFA/Y30 PR8 IFA/170512 Y30 IFA HR-paper Fig/170224 Yusin ekisu H2O 3.125% #1 overlay Fig-bar.tif]

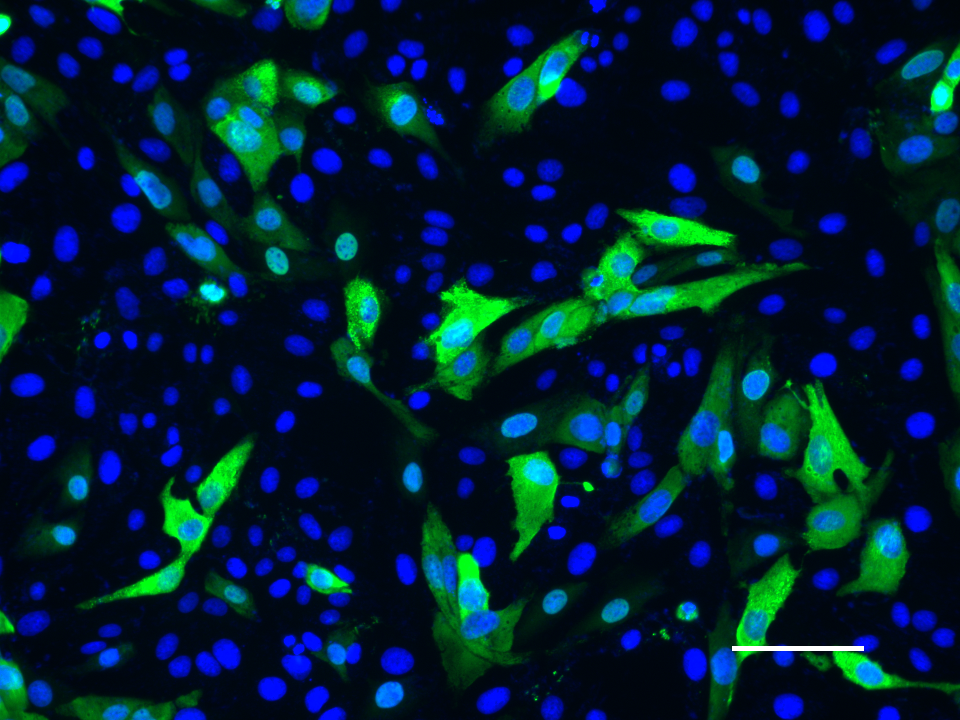

Supplement: S2 Raw data — (ZIP) [file pone.0244885.s002.zip › Y30 Fig. 2/Y30 IFA/Y30 PR8 IFA/170512 Y30 IFA HR-paper Fig/170224 Yusin ekisu H2O 12.5% #2 overlay Fig-bar.tif]

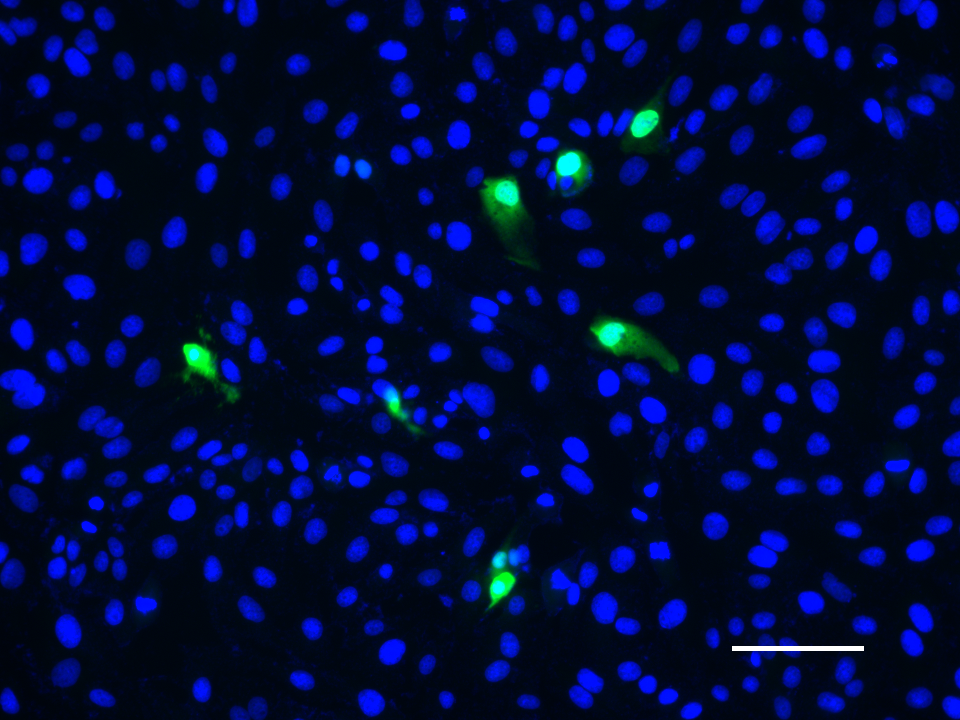

Supplement: S2 Raw data — (ZIP) [file pone.0244885.s002.zip › Y30 Fig. 2/Y30 IFA/Y30 PR8 IFA/170512 Y30 IFA HR-paper Fig/170224 Yusin ekisu Y30 3.125% #2 overlay Fig-bar.tif]

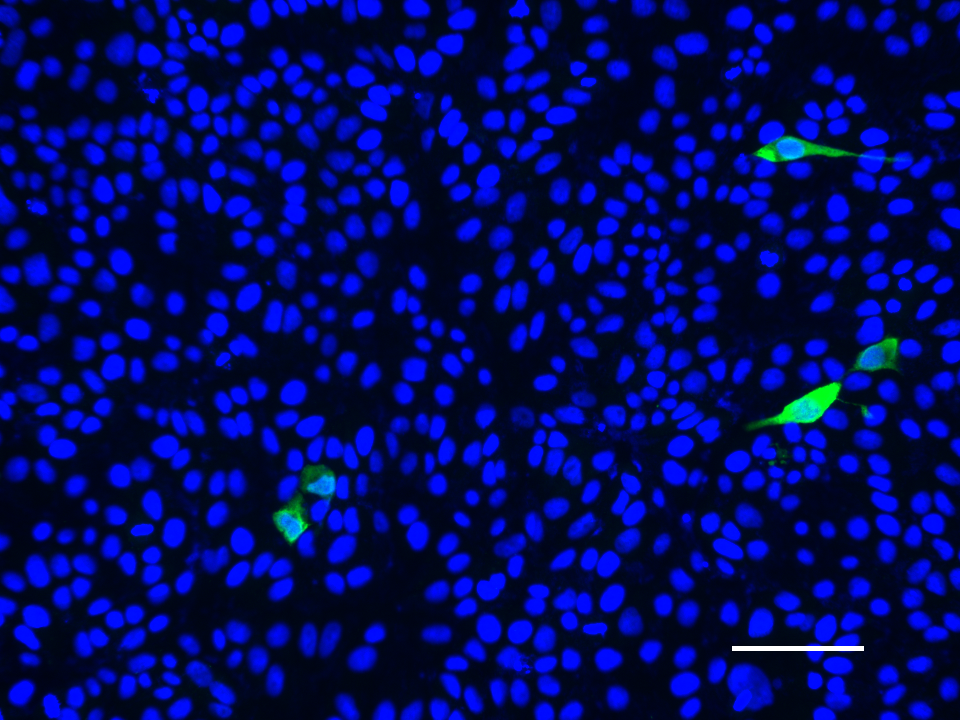

Supplement: S2 Raw data — (ZIP) [file pone.0244885.s002.zip › Y30 Fig. 2/Y30 IFA/Y30 PR8 IFA/170512 Y30 IFA HR-paper Fig/170224 Yusin ekisu bakuchiol 12.5uM #1 overlay Fig-bar.tif]

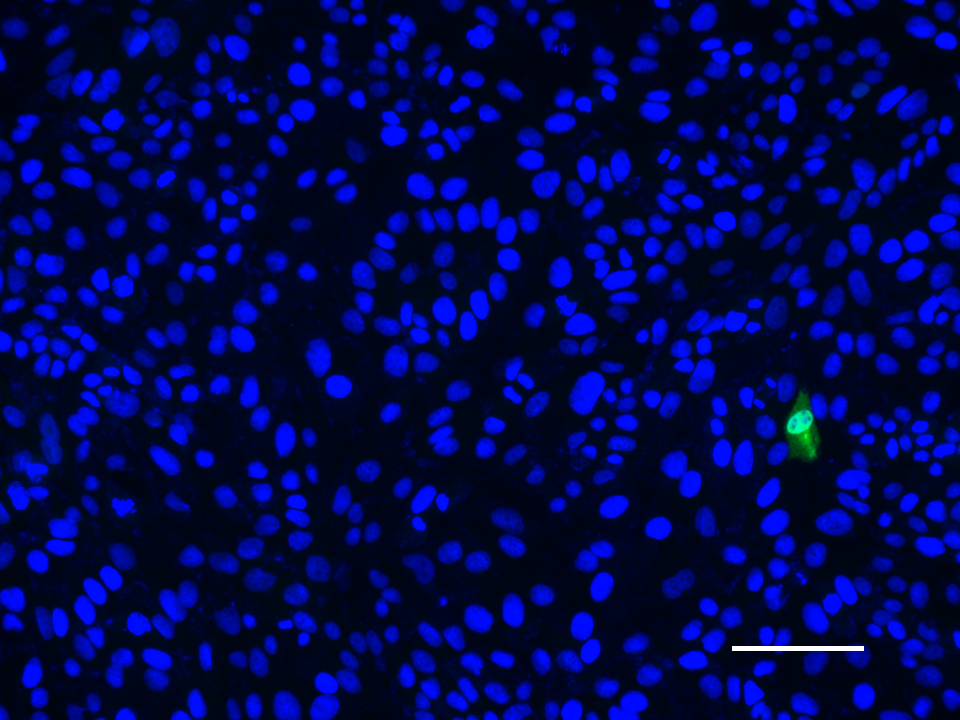

Supplement: S2 Raw data — (ZIP) [file pone.0244885.s002.zip › Y30 Fig. 2/Y30 IFA/Y30 PR8 IFA/170512 Y30 IFA HR-paper Fig/170224 Yusin ekisu Y30 6.25% #1 overlay Fig-bar.tif]

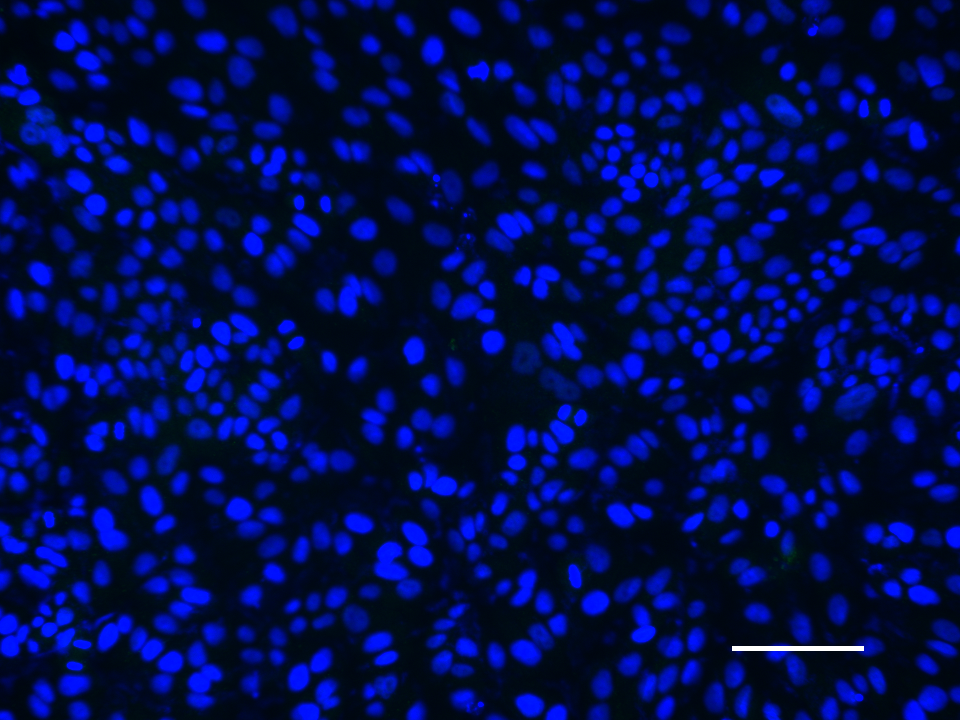

Supplement: S2 Raw data — (ZIP) [file pone.0244885.s002.zip › Y30 Fig. 2/Y30 IFA/Y30 PR8 IFA/170512 Y30 IFA HR-paper Fig/170224 Yusin ekisu Y30 12.5% #2 overlay Fig-bar.tif]

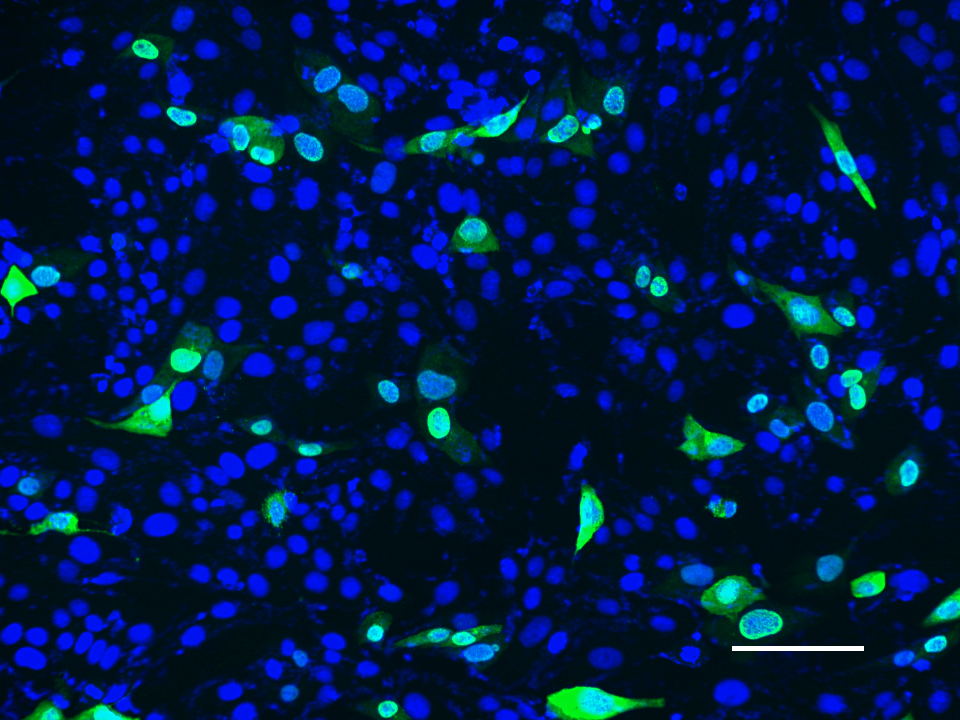

Supplement: S2 Raw data — (ZIP) [file pone.0244885.s002.zip › Y30 Fig. 2/Y30 IFA/Y30 Aichi IFA/180519 Y30 Aichi IFA HR-paper Fig/HR_180519 Yusin ekisu Y30 Aichi IFA MillQ 6.25 n-1 overlay Fig.tif]

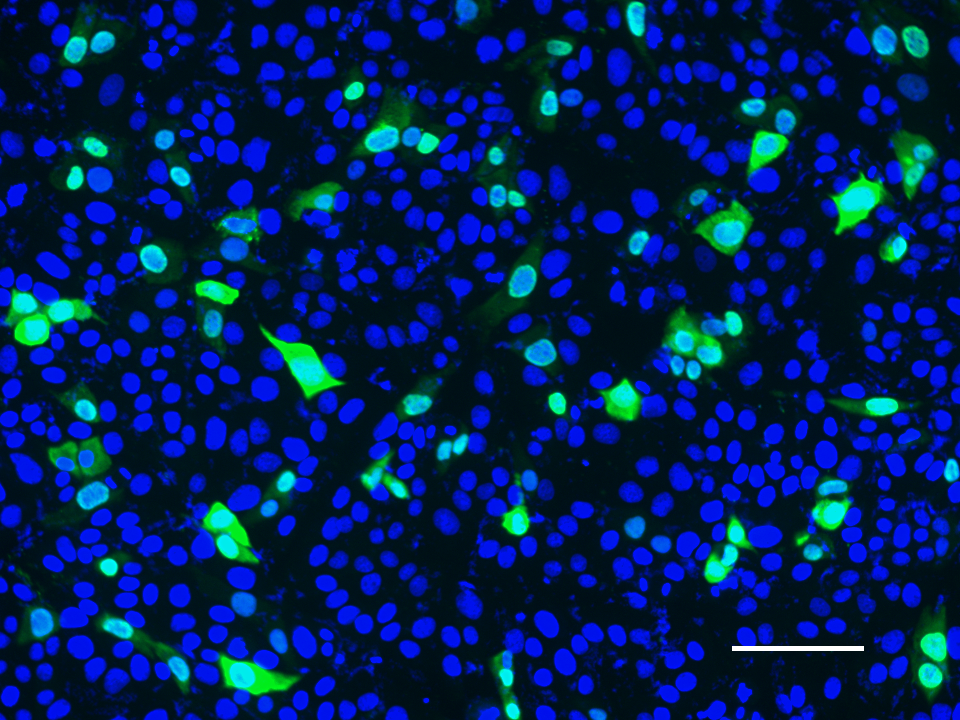

Supplement: S2 Raw data — (ZIP) [file pone.0244885.s002.zip › Y30 Fig. 2/Y30 IFA/Y30 Aichi IFA/180519 Y30 Aichi IFA HR-paper Fig/HR_180519 Yusin ekisu Y30 Aichi IFA MillQ 3.13 n-3 overlay Fig.tif]

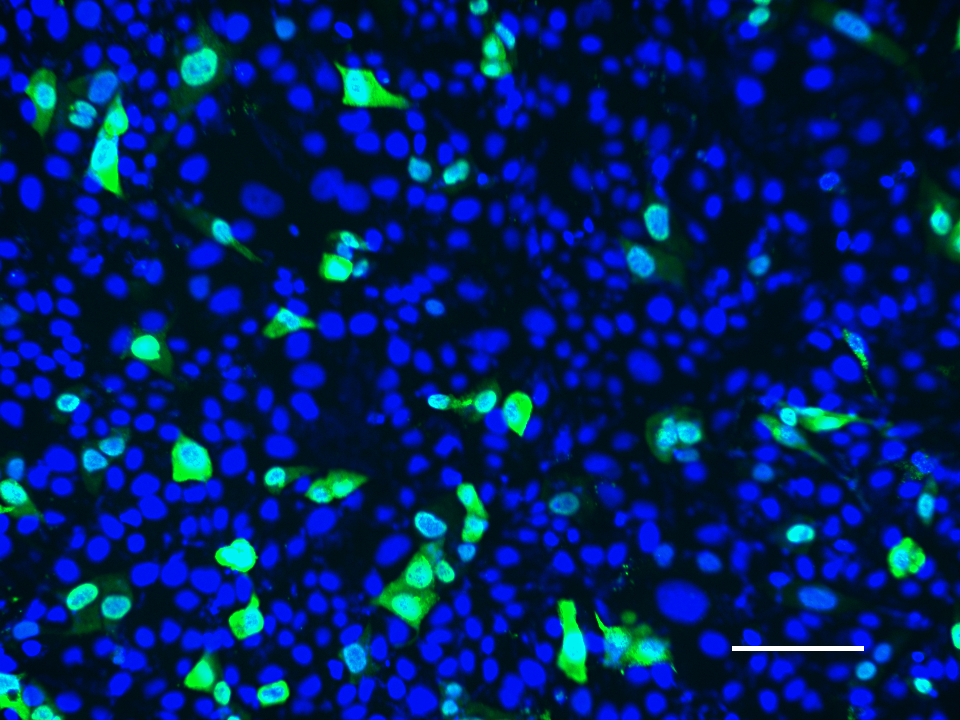

Supplement: S2 Raw data — (ZIP) [file pone.0244885.s002.zip › Y30 Fig. 2/Y30 IFA/Y30 Aichi IFA/180519 Y30 Aichi IFA HR-paper Fig/HR_180519 Yusin ekisu Y30 Aichi IFA Y30 3.13 n-1 overlay Fig.tif]

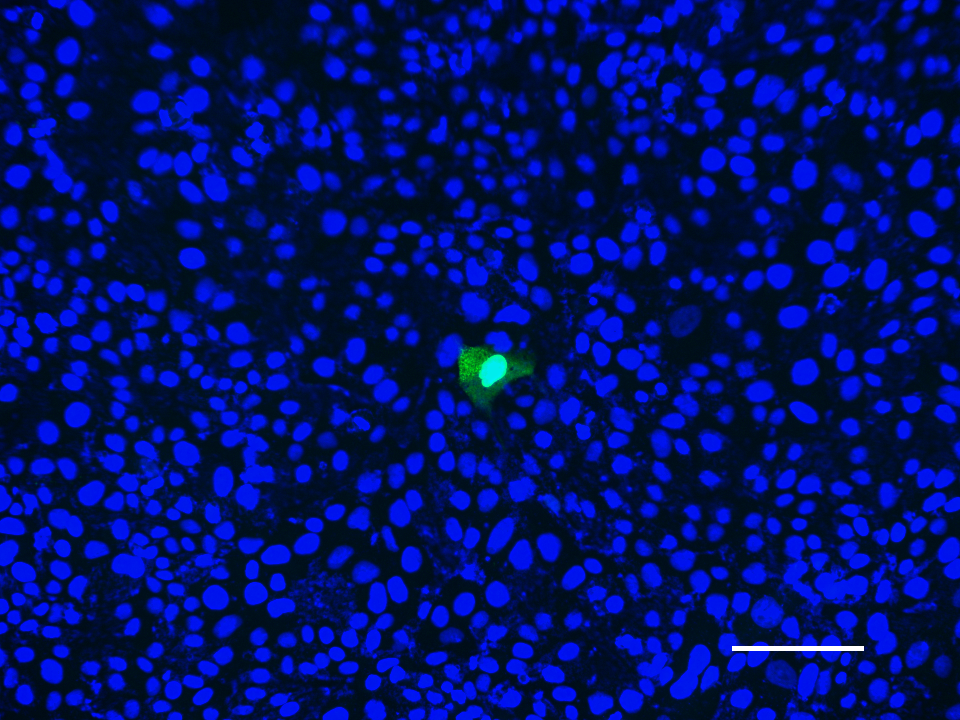

Supplement: S2 Raw data — (ZIP) [file pone.0244885.s002.zip › Y30 Fig. 2/Y30 IFA/Y30 Aichi IFA/180519 Y30 Aichi IFA HR-paper Fig/HR_180519 Yusin ekisu Y30 Aichi IFA Y30 12.5 n-3 overlay Fig.tif]

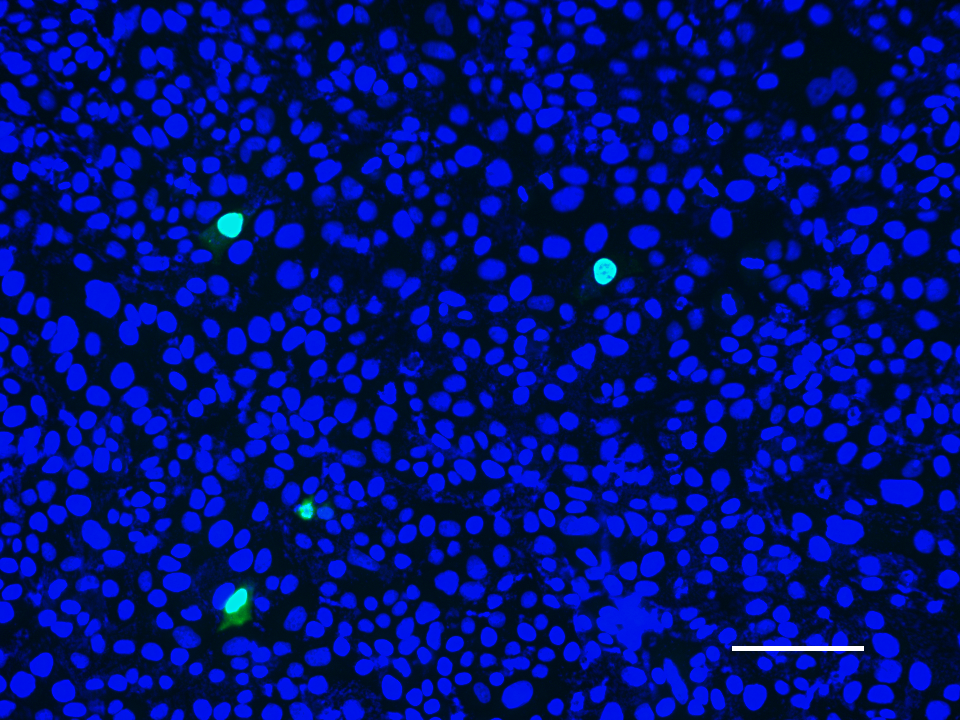

Supplement: S2 Raw data — (ZIP) [file pone.0244885.s002.zip › Y30 Fig. 2/Y30 IFA/Y30 Aichi IFA/180519 Y30 Aichi IFA HR-paper Fig/HR_180519 Yusin ekisu Y30 Aichi IFA Y30 6.25 n-3 overlay Fig.tif]

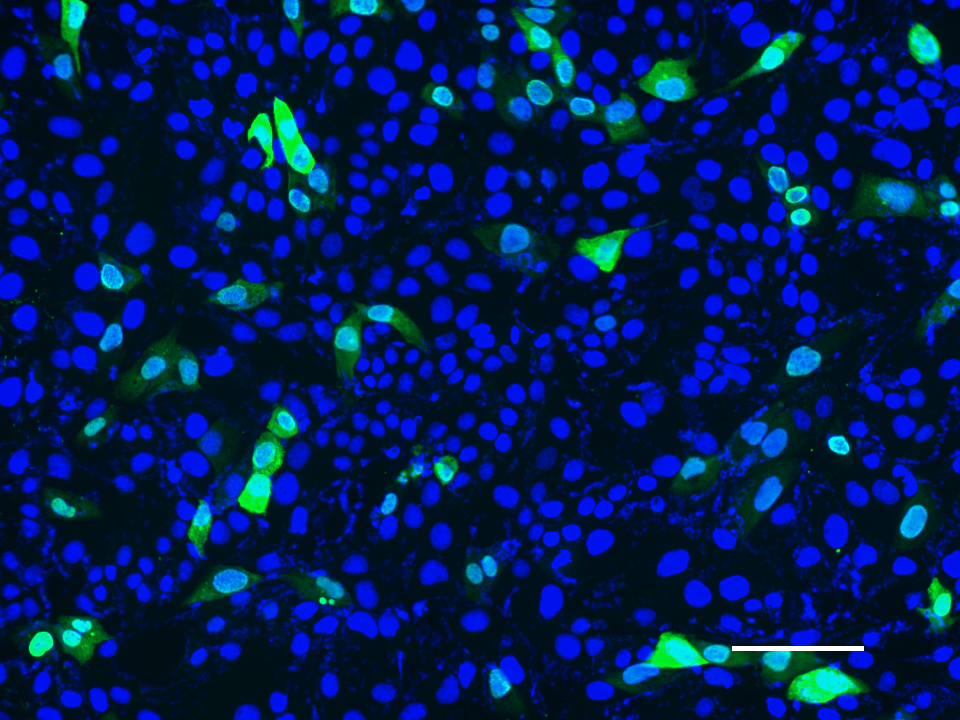

Supplement: S2 Raw data — (ZIP) [file pone.0244885.s002.zip › Y30 Fig. 2/Y30 IFA/Y30 Aichi IFA/180519 Y30 Aichi IFA HR-paper Fig/HR_180519 Yusin ekisu Y30 Aichi IFA MillQ 12.5 n-2 overlay Fig.tif]

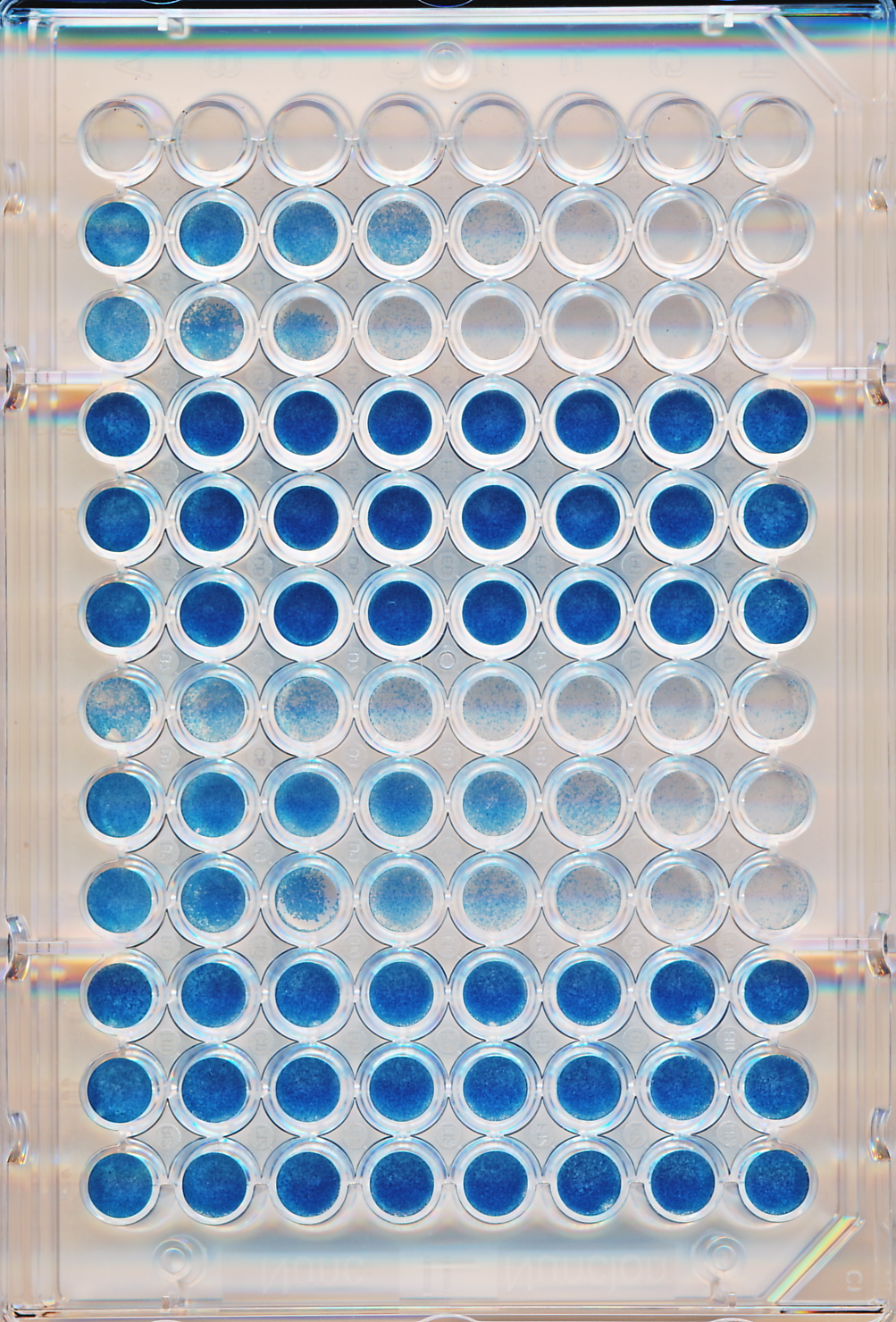

Supplement: S2 Raw data — (ZIP) [file pone.0244885.s002.zip › Y30 Fig. 2/Y30 H1N1 CA NB/170522 Y30 NB Staining CA 5moi,10moi.jpg]

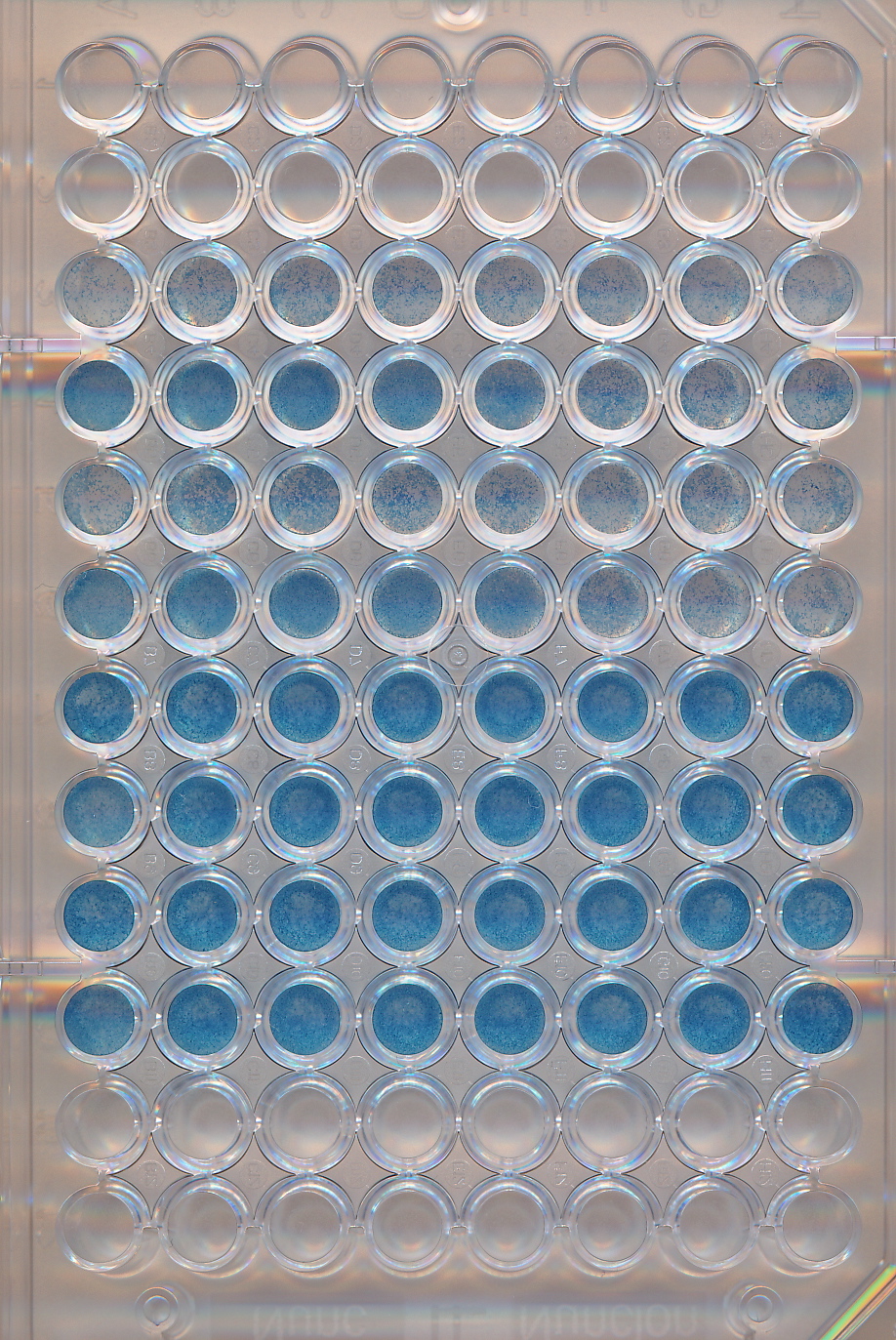

Supplement: S2 Raw data — (ZIP) [file pone.0244885.s002.zip › Y30 Fig. 2/Y30 H3N2 NB/180424 Y30 NB Staining H3N2.jpg]

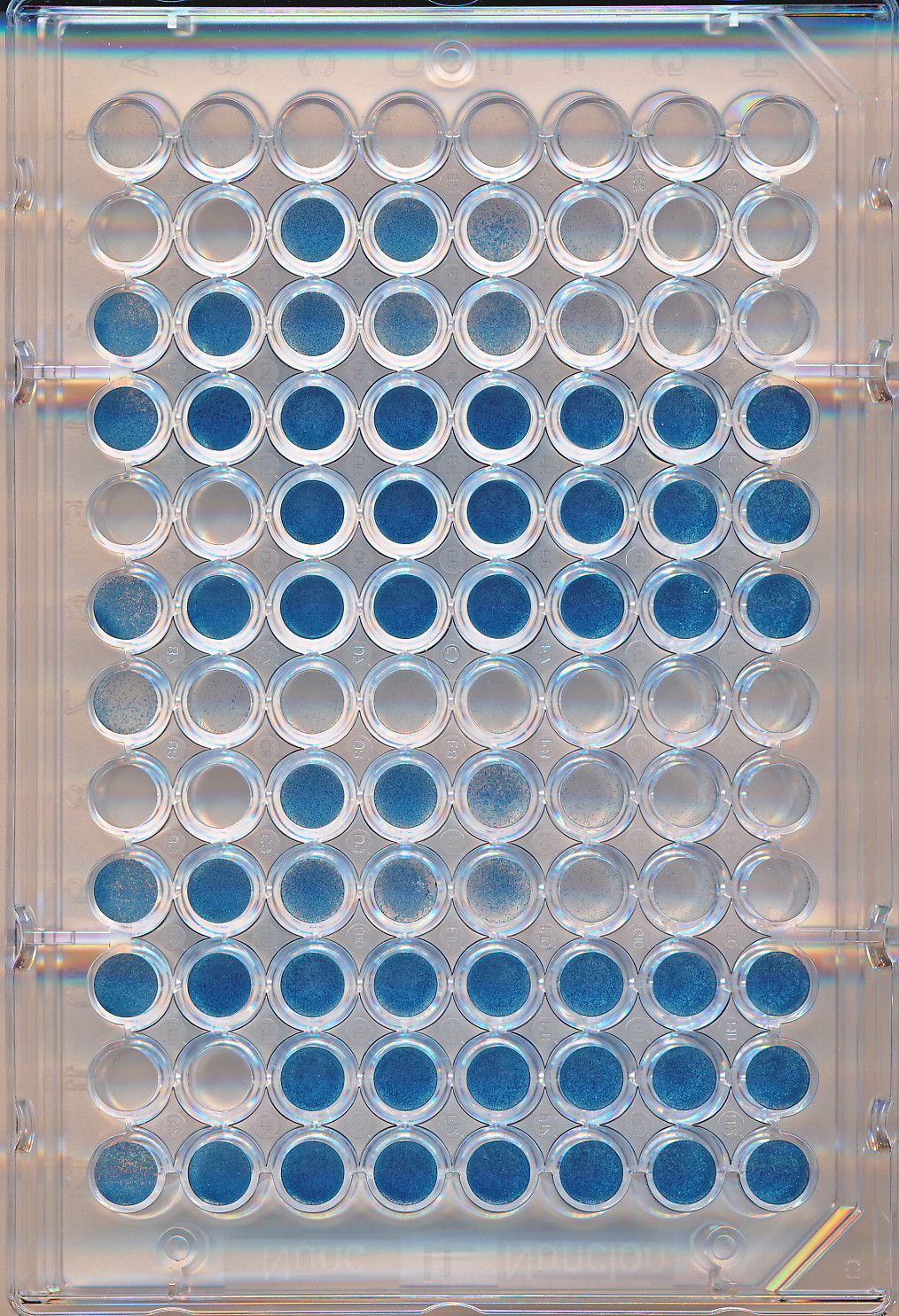

Supplement: S2 Raw data — (ZIP) [file pone.0244885.s002.zip › Y30 Fig. 2/Y30 H1N1 PR8 NB/170322 Y30 NB staining 72h #1,2.jpg]

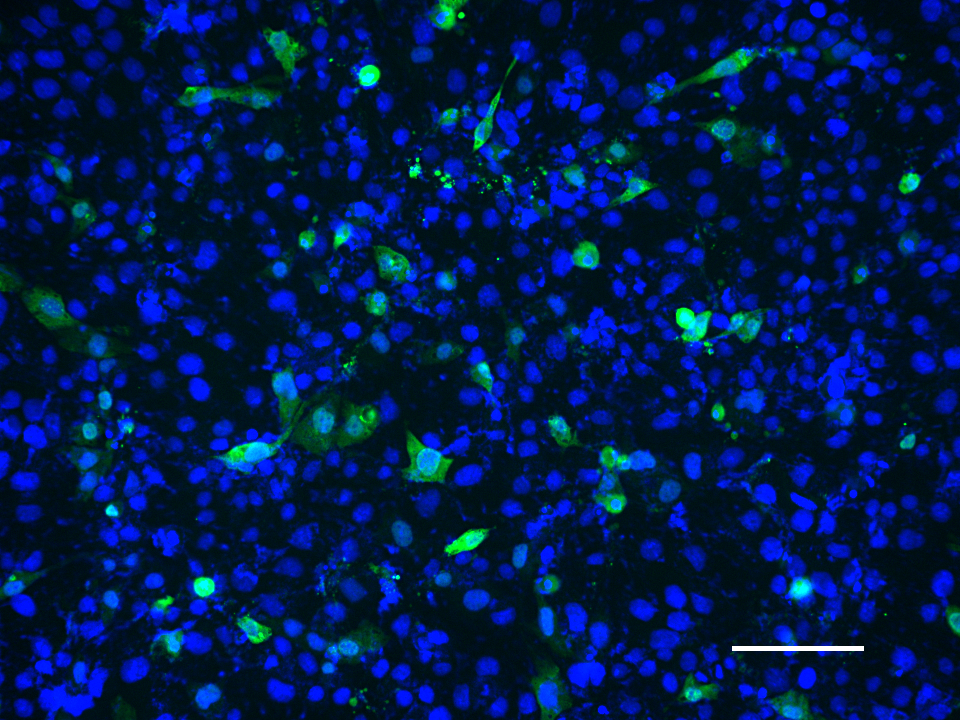

Supplement: S4 Raw data — (ZIP) [file pone.0244885.s004.zip › Figure 4B photo/HR_191003 pre-sample PR8 6.25uM Baku 2.tif]

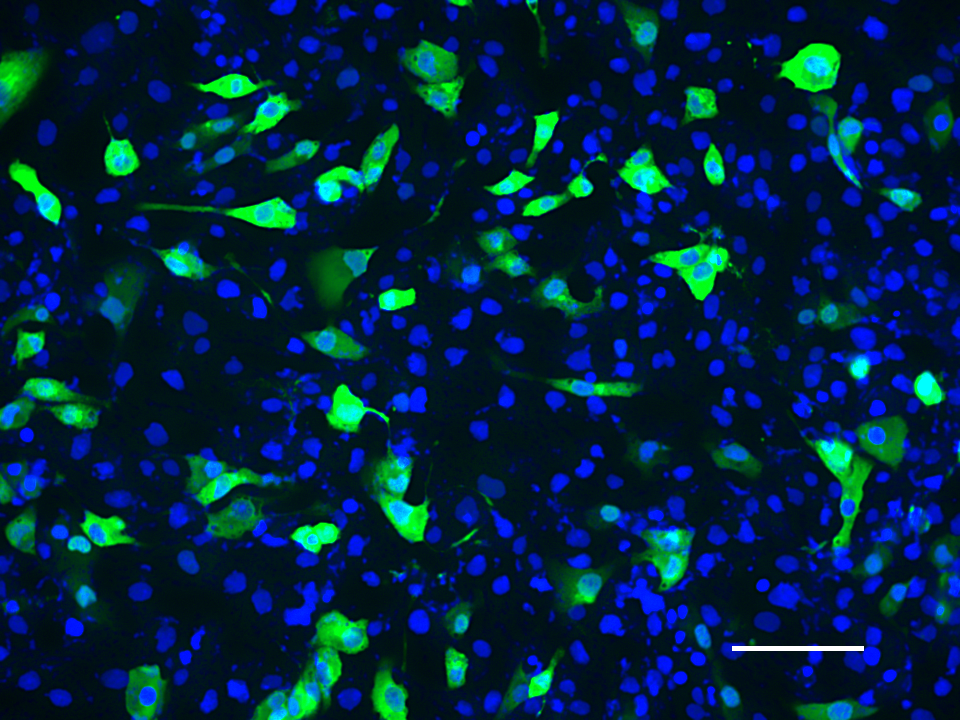

Supplement: S4 Raw data — (ZIP) [file pone.0244885.s004.zip › Figure 4B photo/HR_191003 pre-sample PR8 12.5 Y30 2.tif]

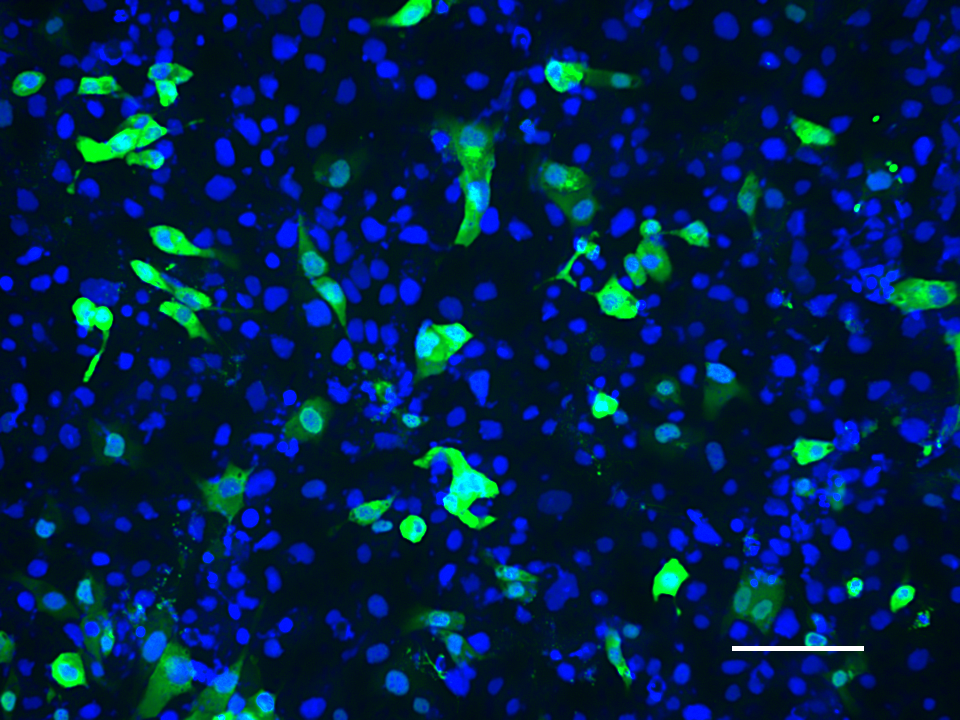

Supplement: S4 Raw data — (ZIP) [file pone.0244885.s004.zip › Figure 4B photo/HR_191003 pre-sample PR8 3.13 Y30 1.tif]

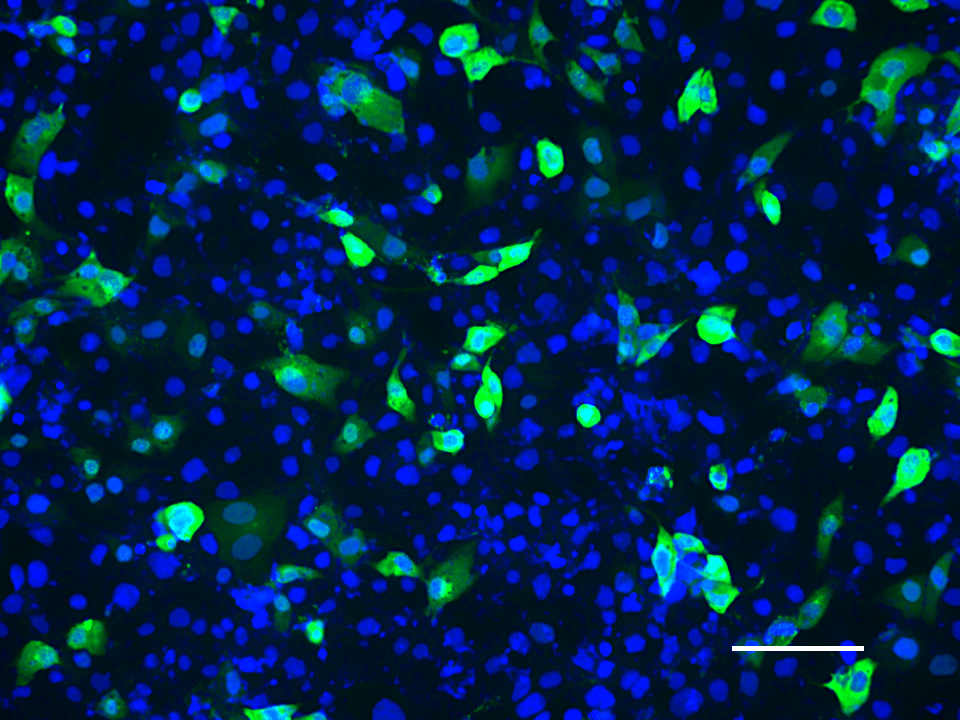

Supplement: S4 Raw data — (ZIP) [file pone.0244885.s004.zip › Figure 4B photo/HR_191003 pre-sample PR8 3.13 DW 4.tif]

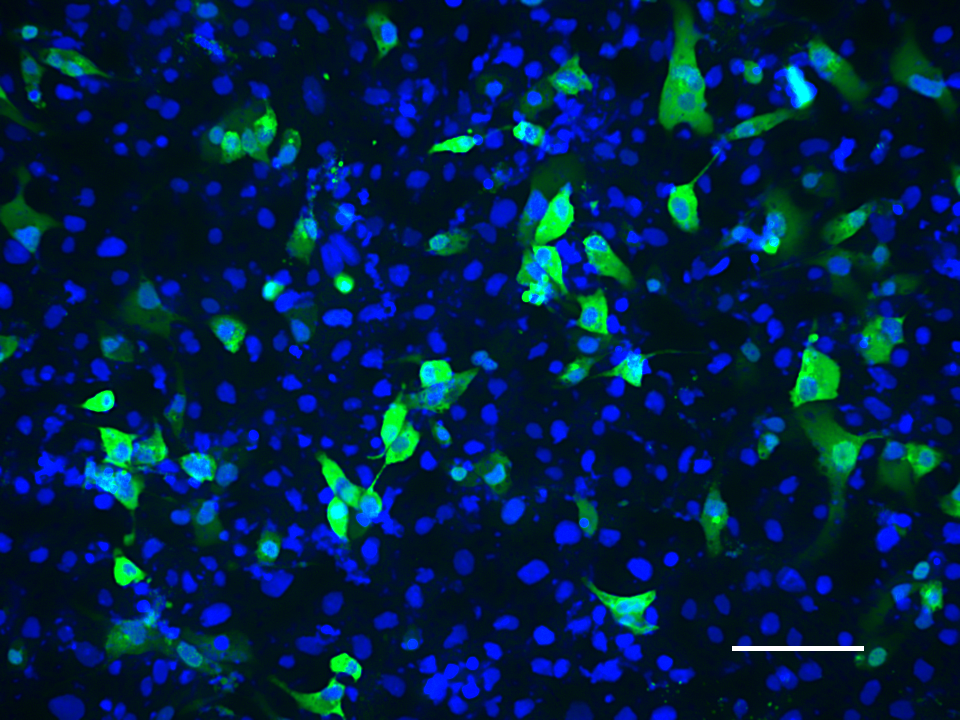

Supplement: S4 Raw data — (ZIP) [file pone.0244885.s004.zip › Figure 4B photo/HR_191003 pre-sample PR8 6.25 Y30 2.tif]

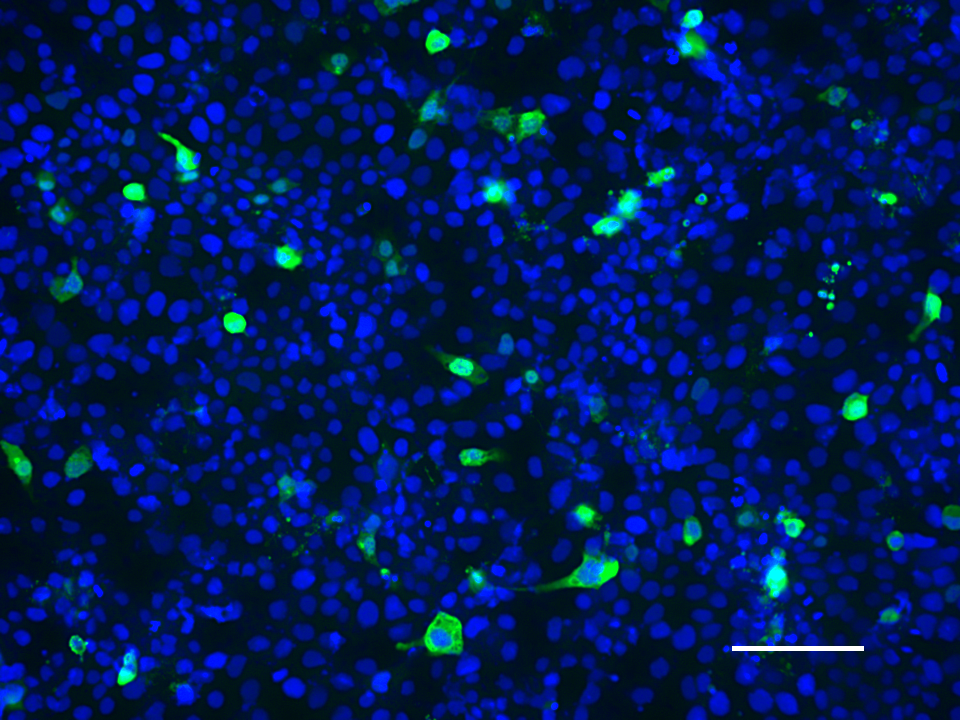

Supplement: S4 Raw data — (ZIP) [file pone.0244885.s004.zip › Figure 4B photo/HR_191003 pre-sample PR8 12.5uM Baku 2.tif]

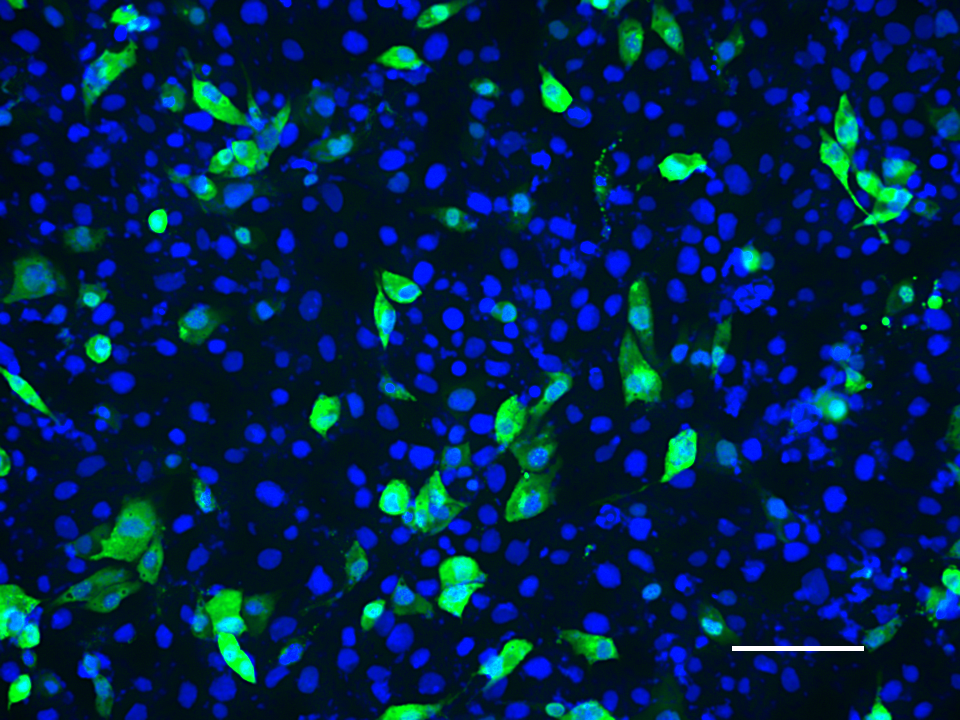

Supplement: S4 Raw data — (ZIP) [file pone.0244885.s004.zip › Figure 4B photo/HR_191003 pre-sample PR8 12.5 DW 2.tif]

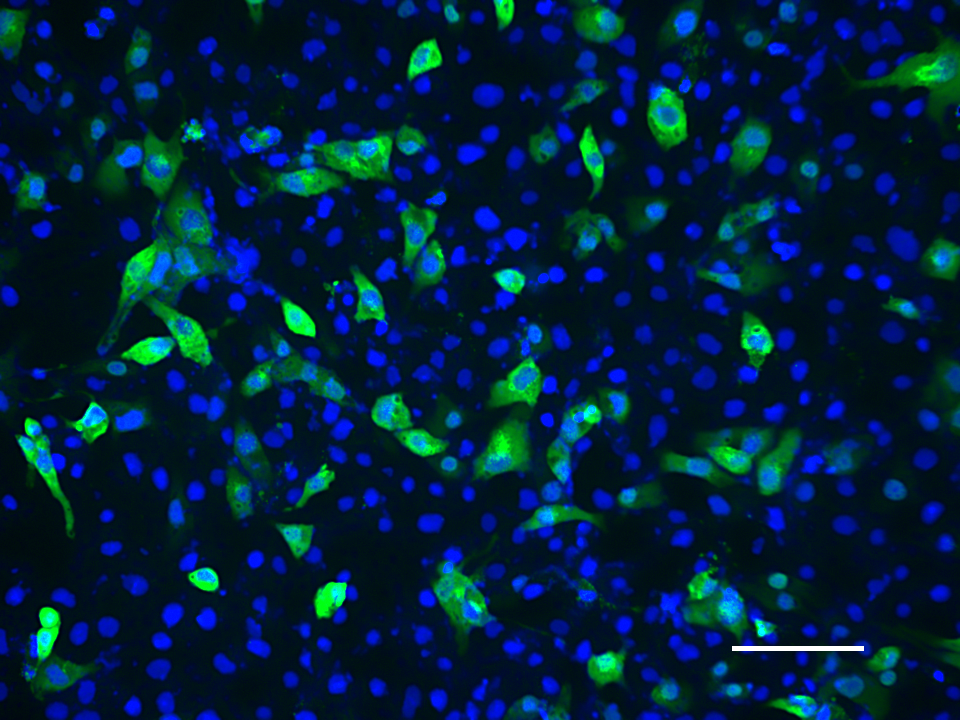

Supplement: S4 Raw data — (ZIP) [file pone.0244885.s004.zip › Figure 4B photo/HR_191003 pre-sample PR8 6.25 DW 2.tif]

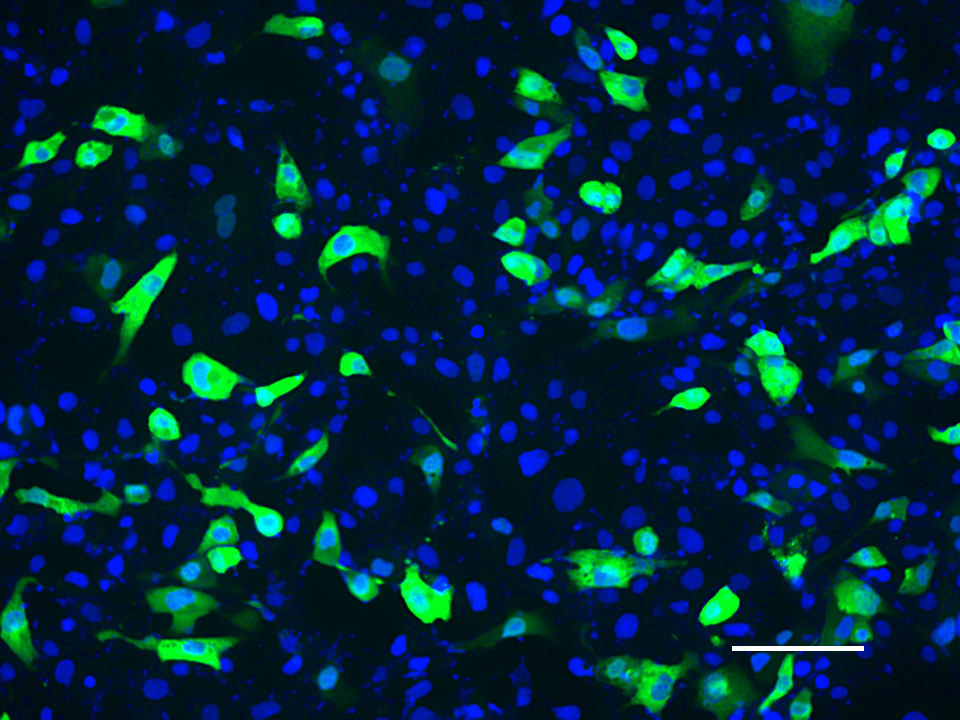

Supplement: S4 Raw data — (ZIP) [file pone.0244885.s004.zip › Figure 4B photo/HR_191003 pre-sample PR8 3.13uM Baku 3.tif]

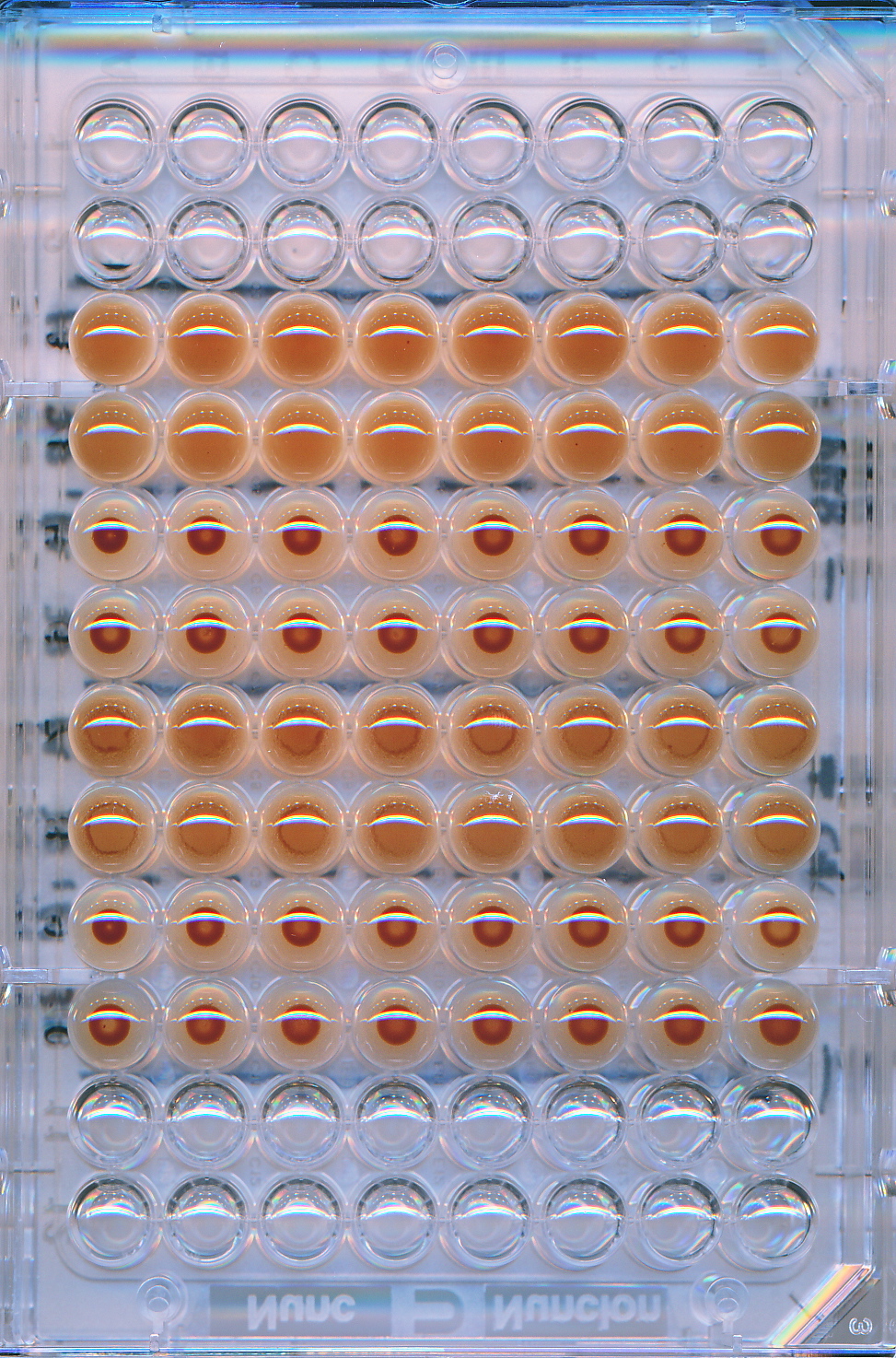

Supplement: S5 Raw data — (ZIP) [file pone.0244885.s005.zip › Y30 Fig. 5/Y30 Fig. 5B/170609 Y30 Hemagglutination inhibition test PR8,CA.jpg]

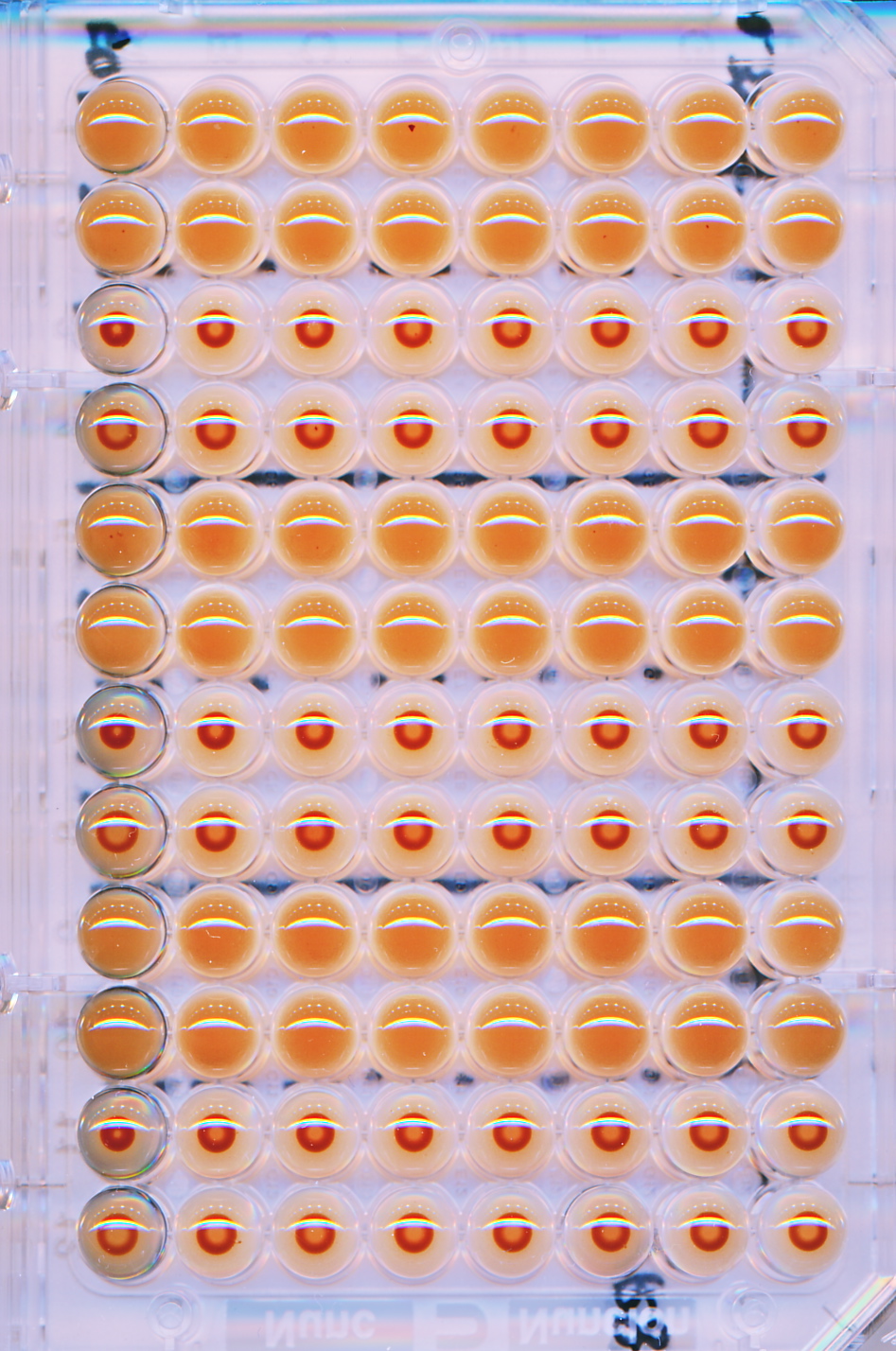

Supplement: S5 Raw data — (ZIP) [file pone.0244885.s005.zip › Y30 Fig. 5/Y30 Fig. 5B/170627 Y30 Hemagglutination inhibition test PR8.jpg]

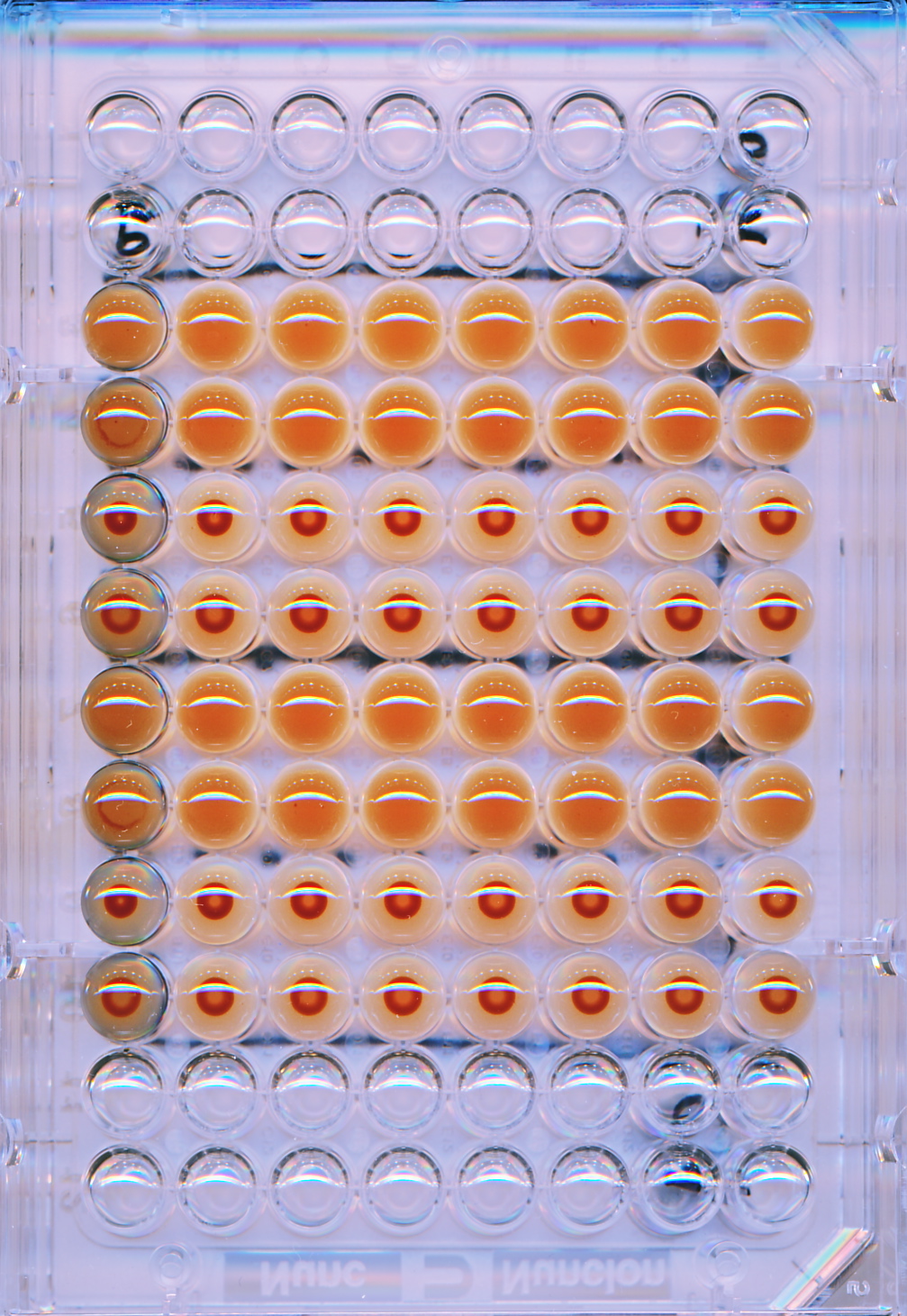

Supplement: S5 Raw data — (ZIP) [file pone.0244885.s005.zip › Y30 Fig. 5/Y30 Fig. 5B/170627 Y30 Hemagglutination inhibition test CA.jpg]

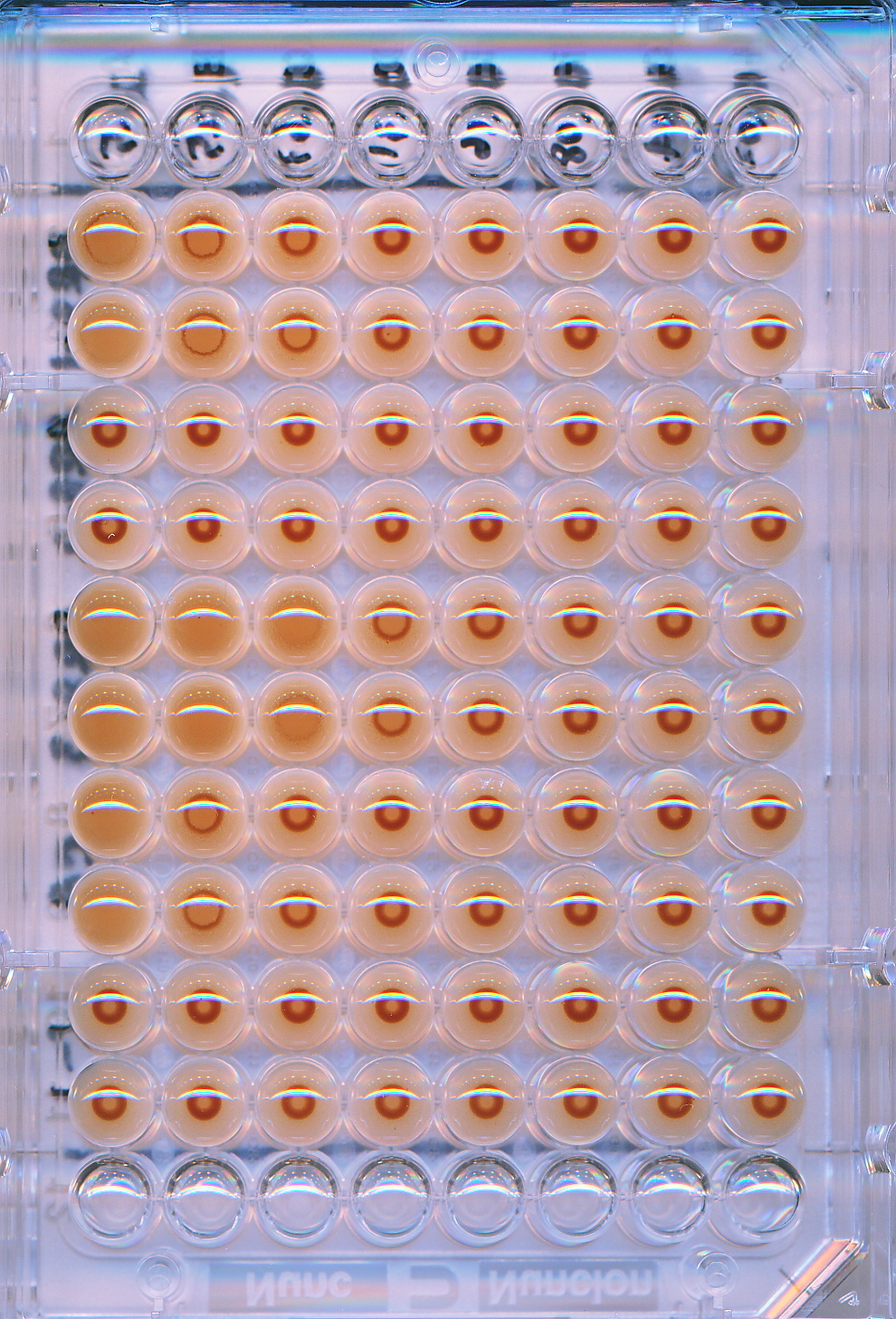

Supplement: S5 Raw data — (ZIP) [file pone.0244885.s005.zip › Y30 Fig. 5/Y30 Fig. 5A/170608 HA assay virusμ┐âσ║aμ▒║σ«Ü.jpg]

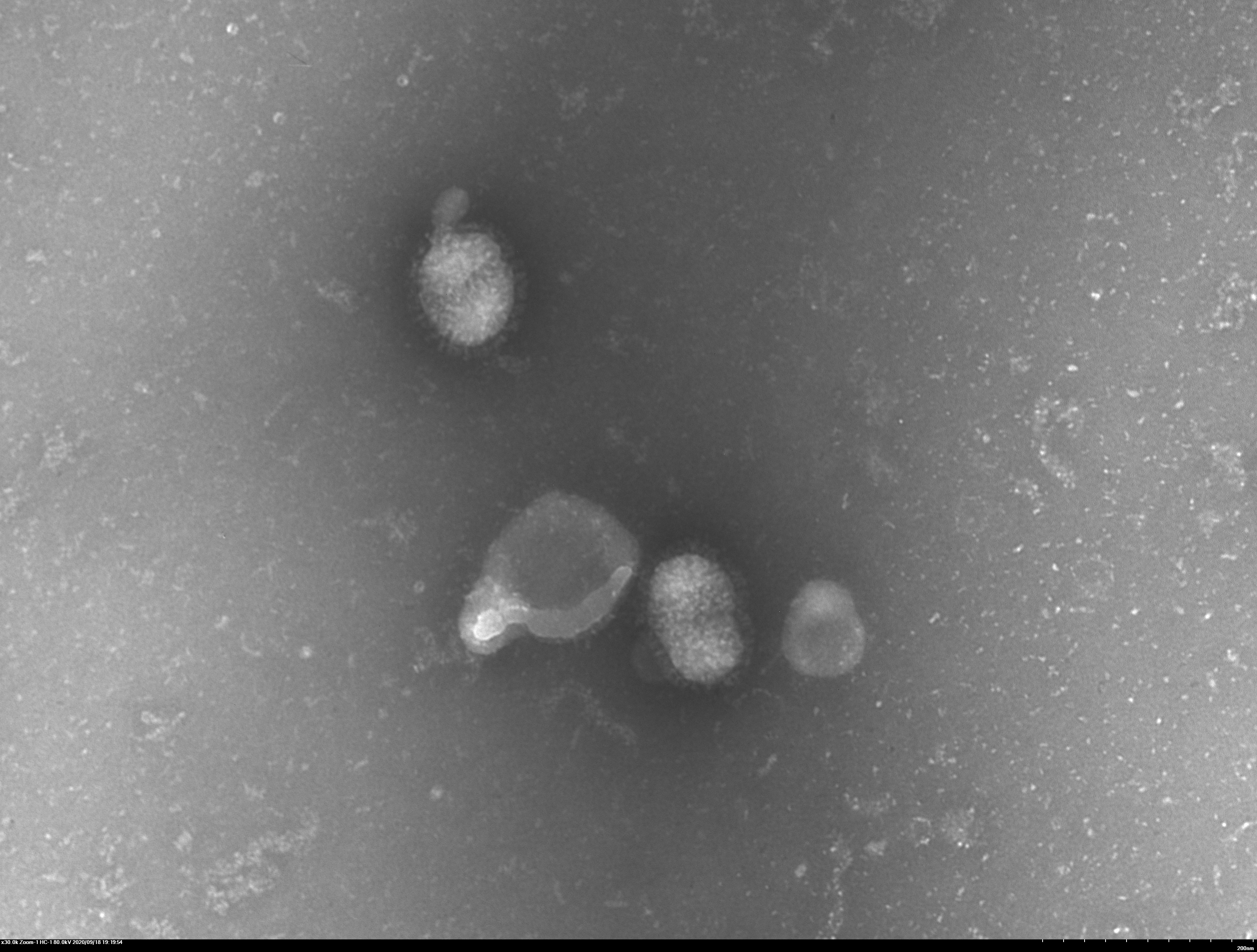

Supplement: S8 Raw data — (ZIP) [file pone.0244885.s008.zip › Y30 Fig. 8/4_Y30_30k_5.tif]

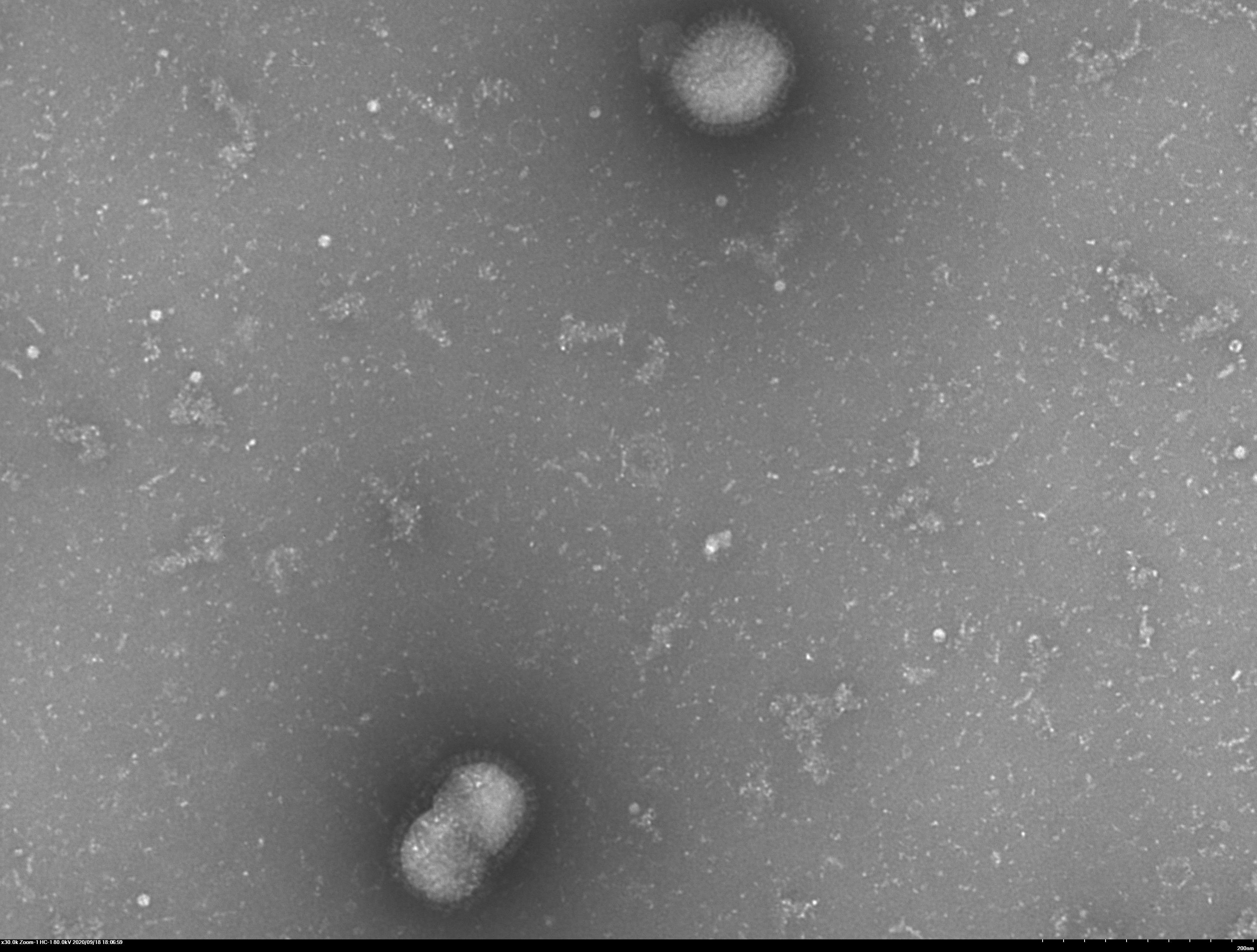

Supplement: S8 Raw data — (ZIP) [file pone.0244885.s008.zip › Y30 Fig. 8/3_water_30k_2.tif]

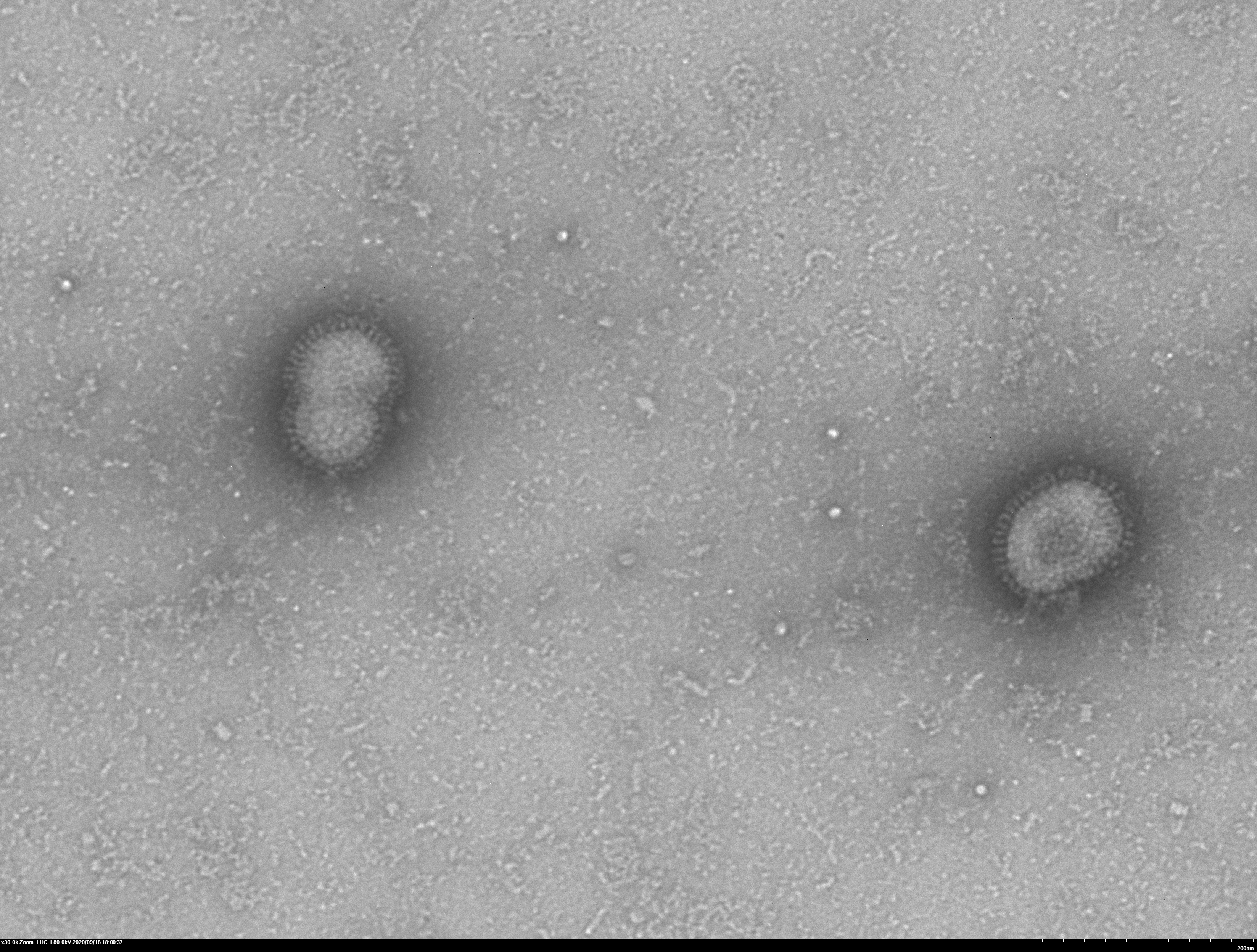

Supplement: S8 Raw data — (ZIP) [file pone.0244885.s008.zip › Y30 Fig. 8/1_PR8_30k_6.tif]

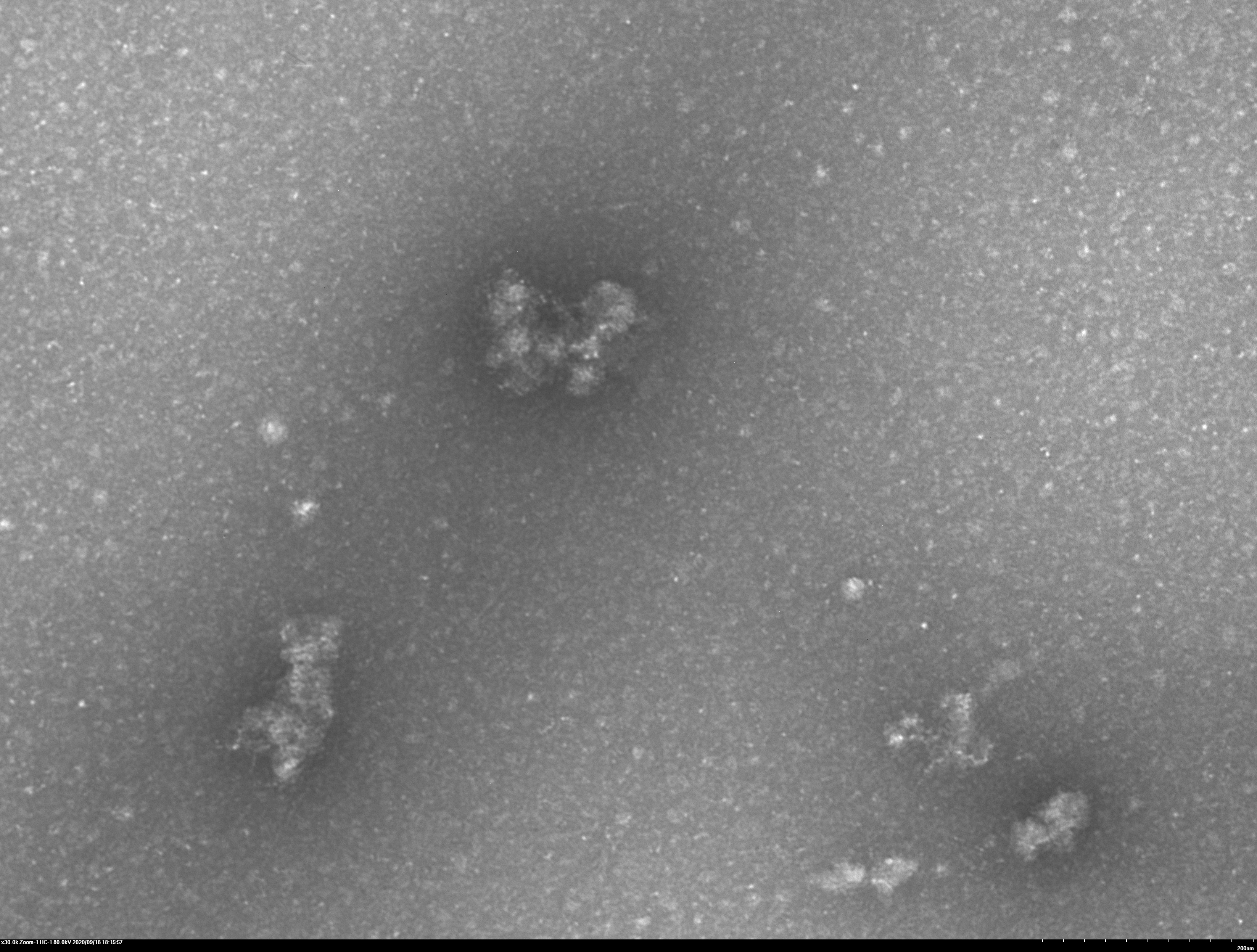

Supplement: S8 Raw data — (ZIP) [file pone.0244885.s008.zip › Y30 Fig. 8/2_CPC_30k_3.tif]

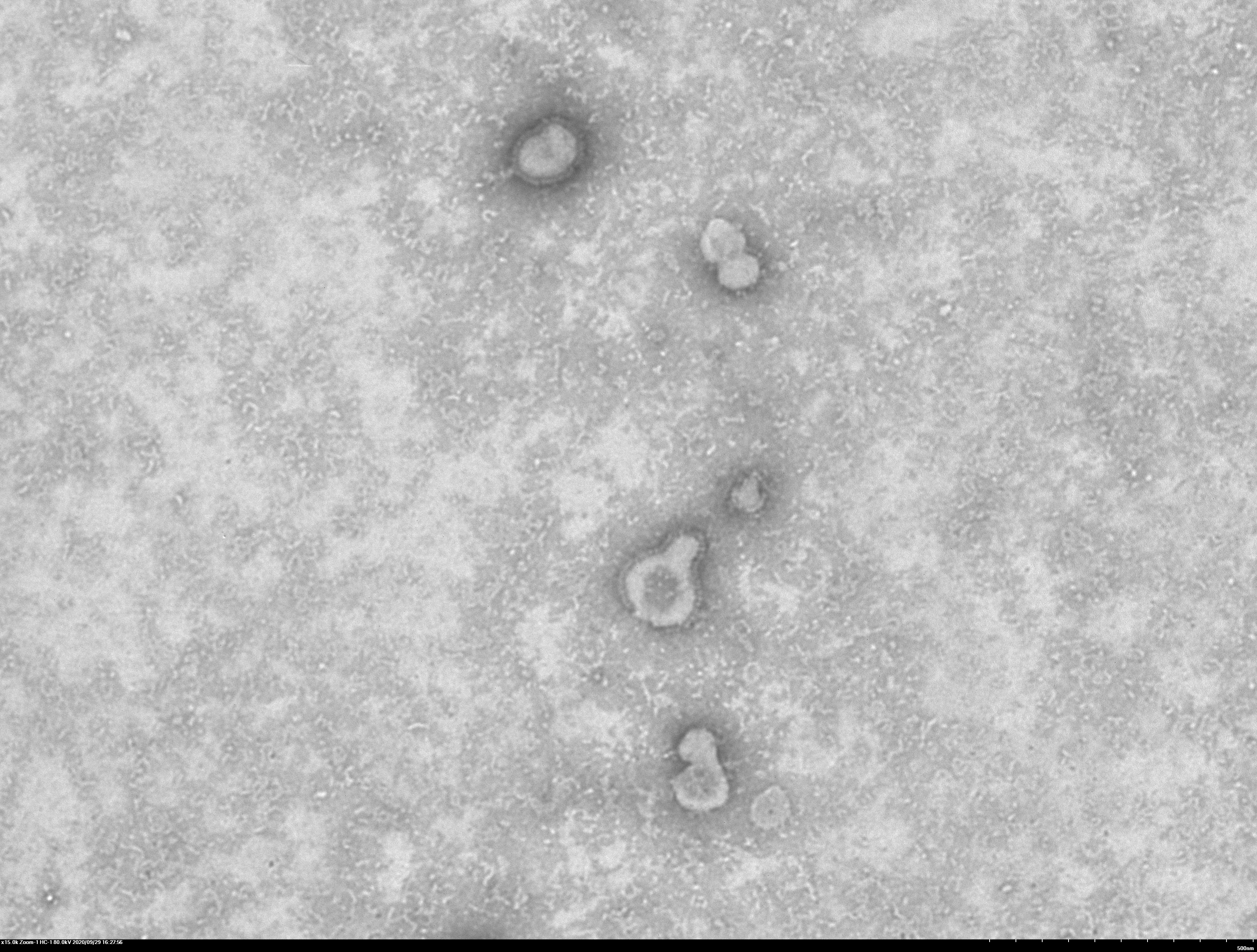

Supplement: S8 Raw data — (ZIP) [file pone.0244885.s008.zip › Y30 Fig. 8/4_Y30_15k_5.tif]

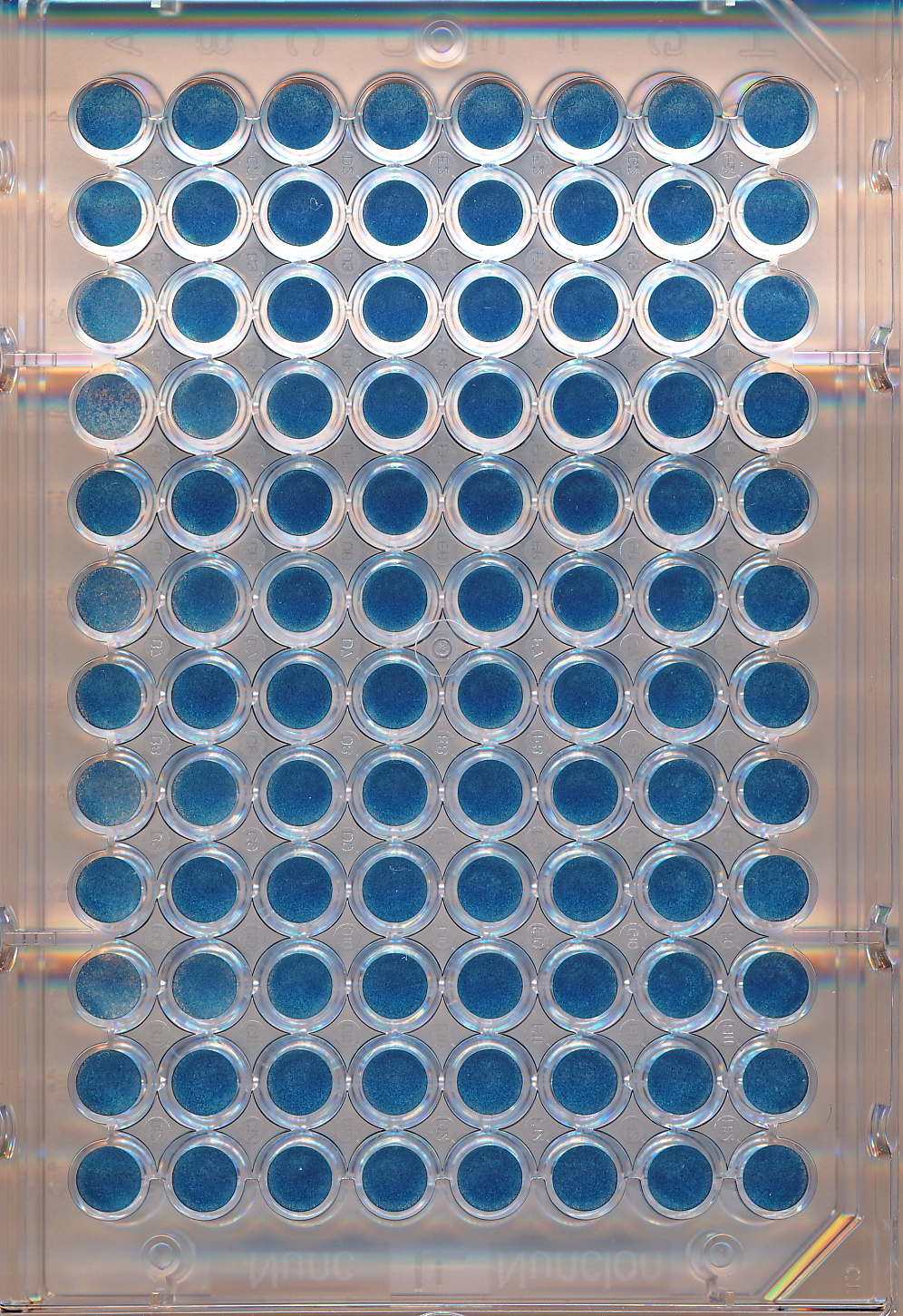

Supplement: S9 Raw data — (ZIP) [file pone.0244885.s009.zip › Y30 Fig. 9/181130 Y30#water EtOAC NB Staining virus-paper.jpg]

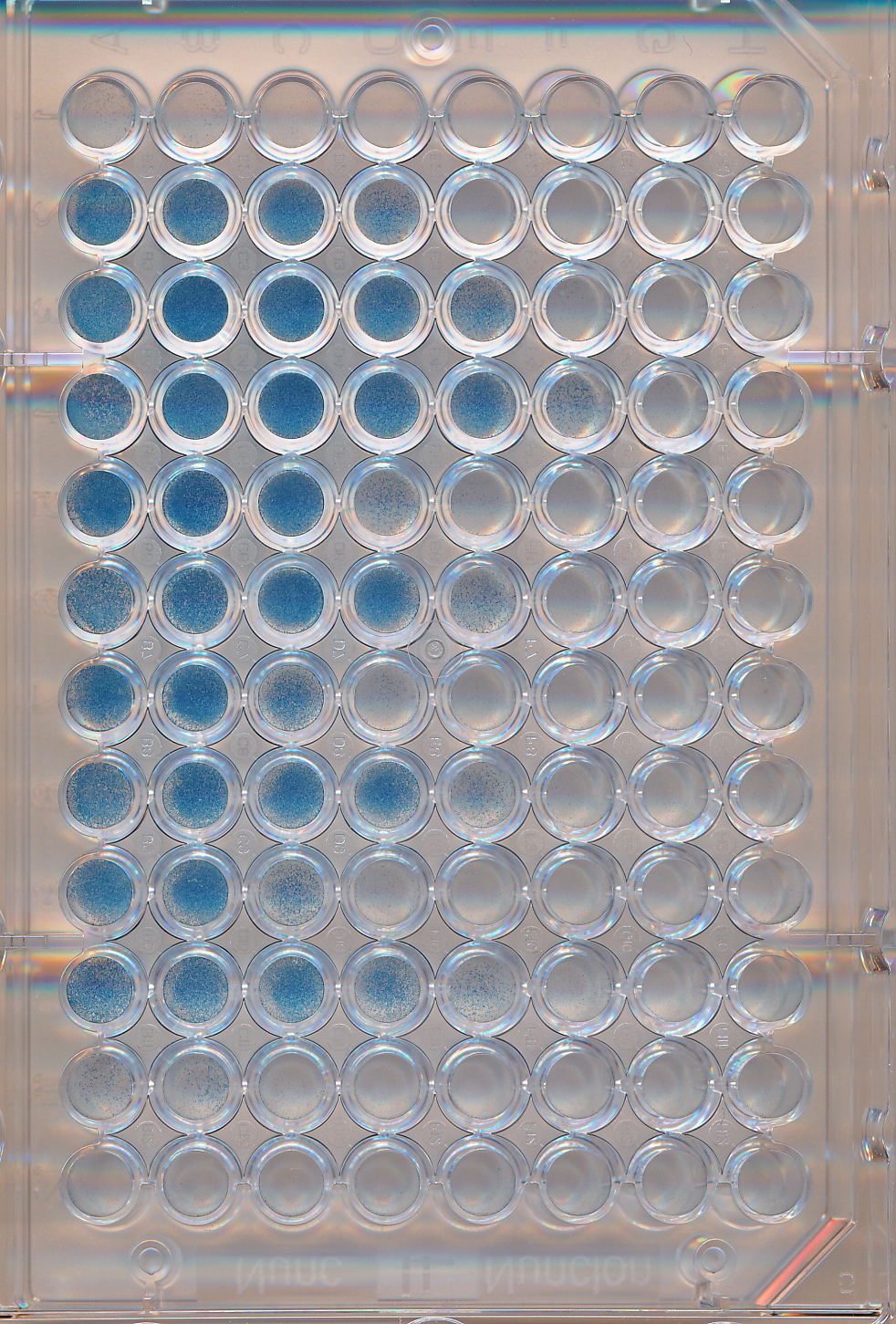

Supplement: S9 Raw data — (ZIP) [file pone.0244885.s009.zip › Y30 Fig. 9/181130 Y30#water EtOAC NB Staining virus+-paper.jpg]
